# Effect of inappropriate complementary feeding practices on the nutritional status of children aged 6-24 months in urban Moshi, Northern Tanzania: Cohort study

**DOI:** 10.1371/journal.pone.0250562

**Published:** 2021-05-13

**Authors:** Rachel Masuke, Sia E. Msuya, Johnson M. Mahande, Ester J. Diarz, Babill Stray-Pedersen, Ola Jahanpour, Melina Mgongo

**Affiliations:** 1 Department of Epidemiology and Biostatistics, Institute of Public Health, Kilimanjaro Christian Medical University College, Moshi, Tanzania; 2 Department of Community Health, Institute of Public Health, Kilimanjaro Christian Medical University College, Moshi, Tanzania; 3 Department of Community Medicine, Kilimanjaro Christian Medical Centre, Moshi, Tanzania; 4 Better Health for African Mother and Child, Moshi, Tanzania; 5 Division of Women, Oslo University Hospital, Rikshospitalet, Norway; 6 Elizabeth Glaser Pediatric AIDS Foundation, Dar es Salaam, Tanzania; Universidade de Sao Paulo Faculdade de Saude Publica, BRAZIL

## Abstract

**Introduction:**

Childhood undernutrition is a major public health problem especially in low and middle-income countries (LMIC). The prevalence of early introduction of complementary feeding, low meal frequency, and low dietary diversity are frequent in LMICs. The effect of inappropriate complementary feeding practices on the nutritional status of children is not well documented in East African countries including Tanzania. Therefore, this study aimed at determining the effect of inappropriate complementary feeding practices on the nutritional status of children aged 6–24 months in urban Moshi, Tanzania.

**Methodology:**

A retrospective cohort study was done using the Pasua and Majengo cohorts of mother-child pairs in urban Moshi who were enrolled from 2002 to 2017. About 3355 mother-child pairs were included in the analysis. Appropriate complementary feeding practices were assessed using WHO IYFP indicators such as age at introduction of solid, semi-solid, or soft foods, minimum dietary diversity, and minimum meal frequency. Nutritional status (stunting, wasting, and underweight) was determined. Multilevel modeling was applied to obtain the effect of inappropriate complementary feeding practices on the nutritional status of children and to account for the clustering effect of mothers and children and the correlation of repeated measures within each child.

**Results:**

Majority of the children (91.2%) were given soft/semi-solid/solid foods before six months of age, 40.3percent had low meal frequency, and 74percent had low dietary diversity. Early introduction of complementary food at age 0–1 month was statistically significantly associated with higher risks of wasting and underweight (ARR 2.9, 95%CI 1.3–6.3; and ARR 2.6, 95% CI 1.3–5.1 respectively). Children with low minimum meal frequency had higher risks of stunting, wasting, and underweight (ARR 2.9, 95%CI 2.3–3.6; ARR 1.9, 95%CI 1.5–2.5 and ARR 1.9, 95%CI 1.5–2.4 respectively). Children with low minimum dietary diversity were more likely to be stunted than is the case with their peers who received the minimum dietary diversity (ARR 1.3, 95% CI 1.01–1.6).

**Conclusion:**

There were a high proportion of children, which were fed inappropriately; Inappropriate complementary feeding practices predisposed children to undernutrition. Our study supports the introduction of complementary feeding, providing minimum dietary diversity, and minimum feeding frequency at six months of age as important in improving the nutritional status of the children.

## Introduction

An appropriate diet is a critical component for proper growth and development of children. Appropriate child feeding practices in the first two years of life provide a critical window of opportunity for the promotion of optimal growth and development [1]. The aspects of Infant and Young Child Feeding Practices, which are important in the first 2 years of life include early initiation of breastfeeding, exclusive breastfeeding, and timely and safe introduction of complementary feeding with continued breastfeeding up to two years of age or beyond [2]. It has been estimated that appropriate complementary feeding practices contribute to 17 percent reduction in the prevalence of stunting at 24 months of age and could avert 6 percent of under-five deaths each year [3, 4]. Suboptimal breastfeeding practices and infectious diseases are the main immediate causes of undernutrition in the first two years of life [5].

WHO recommends an infant to be exclusively breastfed for the first six months of life, then begin nutritionally adequate, safe, and appropriately-fed complementary foods from six to 24 months in order to meet the evolving needs of the growing infant [2]. However, few children under two years of age are fed appropriately. Globally, it has been estimated that only 41percent of infants are exclusively breastfed for six months, 25 percent receive the minimum dietary diversity (MDD) and 51 percent receive the minimum required meal frequency (MMF) [6]. The highest burden of inappropriate complementary feeding practices are in low and middle-income countries [7]. Data from a recent Tanzania Demographic and Health Survey showed that 59 percent of infants are exclusively breastfed for six months, 39 percent of children aged 6–24 months are given minimum recommended meal frequency and 26 percent are given minimum recommended diverse diet [8].

Despite the major progress in reducing the prevalence of child undernutrition, the burden is still high in LMICs [9]. Globally, estimates show that 149 million of under-five children are stunted and 49 million of under-five children are wasted. Low and middle-income countries contribute to 65 percent of the stunted children and 73 percent of wasted children [10]. In Tanzania, according to the Tanzania Demographic Health Survey stunting has been declining from 48 percent in 2000 to 34 percent in 2015 (equal to 0.9% annual decrease), underweight decreased from 24 to 14 percent while wasting remains at 5 percent in the same period [8]. This reveals a slow progress towards reaching global nutrition targets of reducing stunting by 40 percent in the year 2025.

Findings of the relationship between complementary feeding practices and child nutritional status remain inconsistent [11]. Child undernutrition is still a significant public health problem and poor complementary feeding is a common practice for many mothers in East African countries including Tanzania [12, 13]. However, the effect of inappropriate complementary feeding practices on the child nutritional status remains unknown. Therefore, this study aims at determining the effect of inappropriate complementary feeding practice on the nutritional status of children. This may be an important aspect in devising strategies of influencing the promotion of various nutrition interventions of reaching this most vulnerable population.

## Methodology

### Data source

This study was a part of a larger cohort established in 2002. Women recruited in the parent study were from three distinct groups. The first group was recruited from 2002 to 2004, the second group was recruited from 2005 to 2013, and the last group was recruited from 2014 to 2017. The main objective of the parent study was to investigate HIV and Sexually Transmitted Diseases among pregnant women in Kilimanjaro [14]. The study included pregnant women who were in their third trimester attending the antenatal care clinic at Pasua and Majengo health centers in Moshi municipality, Kilimanjaro region. These two clinics were selected because they have the largest number of clients and represent women from the largest geographical areas in urban Moshi [8]. Details of the methodology are documented elsewhere and can be obtained from the previous studies [15–20].

Baseline data were collected during recruitment using a questionnaire designed to collect information regarding maternal socio-demographic data, obstetric history, and behavioral and employment status. Women and their children were followed up after delivery and each mother-child pair was seen at the health centers after 3 months until the child turns 2 years of age and thereafter a follow-up was after every 6 months until the child turns 5 years of age. Four trained research assistants (two in each health facility) were taking anthropometrics and collecting child feeding practices using a child questionnaire that was adopted from WHO indicators for assessing IYCF practices [21]. This was modified to capture common foods consumed in Moshi, Tanzania at each visit. Anthropometric measurements (height and weight) were taken using the recommended WHO procedures [22]. The recumbent length (for children below 24 months) was taken using a local made flat wood stadiometer. Weight of undressed children was taken using SECA 813 digital scale (SECA, GmbH &Co.KG, Hamburg, Germany) and the length and weight of children were taken to the nearest 0.1cm and 0.1g.

### Study design and setting

**Fig 1:**
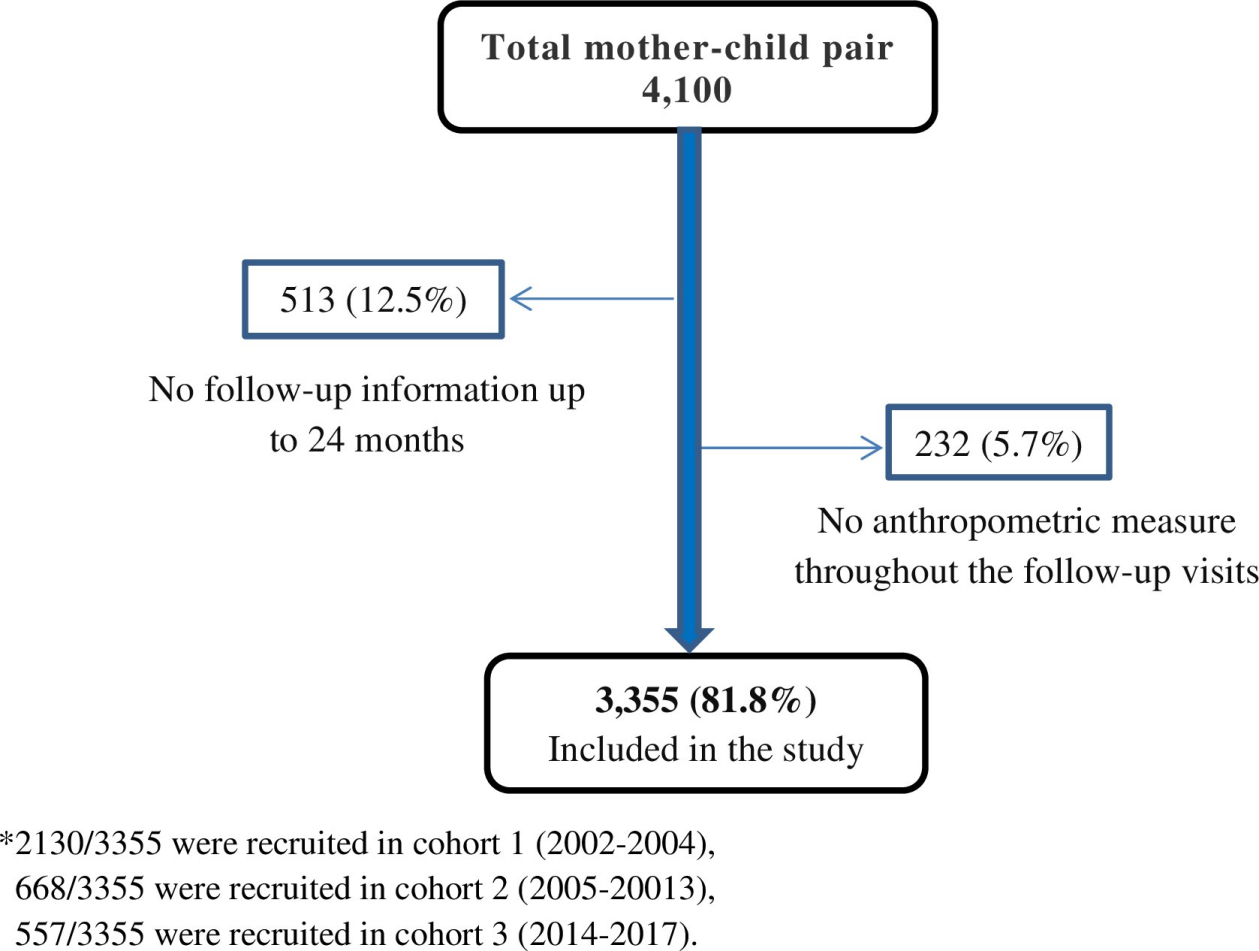
Flow chart for selecting mother-infant pairs to be included in the study.

This was a retrospective cohort study, which was carried out using data from Pasua, and Majengo cohorts, which are health facilities located in the urban Moshi, Kilimanjaro region. About 3355 mother-child pairs from 2002 to 2017 were included in this analysis. The following were the inclusion criteria: mother-child pairs with any follow-up visit from 6 to 24 months having anthropometrics and feeding data (Fig 1).

### Variables measurements

#### Dependent variables

Dependent variables were stunting [height-for-age (HAZ)], wasting [weight-for-height (WHZ), and underweight [weight-for-age (WAZ)]. Children were classified as stunted, wasted or underweight if their Z-scores were <-2 Standard Deviation (SD) [23].

#### Independent variables

Inappropriate complementary feeding practices were defined as the introduction of complementary foods that fails to meet either of the three indicators of time of introduction (six months of age), minimum meal frequency or minimum dietary diversity [1]. Therefore, to assess inappropriate complementary feeding practices, three primary independent variables were used, which were the introduction of solid, semi-solid, or soft foods, minimum dietary diversity, and minimum meal frequency. Each of these indicators was measured separately.

Introduction of solid, semi-solid, or soft foods was measured as the age of a child in months when soft foods/semi-solid foods/solid foods were introduced. Mothers/caregivers reported when a child started receiving soft-foods, semi-solids, and solid foods. Introduction of solid, semi-solid, or soft foods was further categorized as early introduction of complementary feeding (grouped as 0–1 month, 2-3months, 4–5 months), and appropriate age of introduction of complementary feeding (as 6–8 months of age) [21].

The 7 food groups were used for the calculation of WHO minimum dietary diversity indicator, which are (i) grains, roots and tubers, (ii) legumes and nuts, (iii) dairy products, (iv) flesh foods, (v) eggs, (vi) vitamin A-rich fruits and vegetables, and (vii) other fruits and vegetables. The dietary diversity score ranged from 0 if none of the food groups is consumed to 7 if all the food groups are consumed. Minimum dietary diversity was constructed from the dietary diversity score using the WHO recommended cut-off point with a value of “1” if the child had consumed four or more groups of foods (minimum dietary diversity) and “0” if the child consumed less than four food groups (low dietary diversity). MDD was estimated during 6, 9, 12, 15 and 24 months visit by asking mothers/caregivers to report the food and liquid consumed during the previous day of the visit as per the WHO guidelines.

Minimum meal frequency was a self-reported question on the number of times per day a child received complementary foods on the day before the visit. MMF was estimated at each visit and a child was judged to have taken an adequate number of meals if he/she received at least the minimum frequency of feeding. This means, 2 times for 6–8 months and 3 times for 9–11 months, 3 times for children aged 12–23 months for breastfed children and 4 times for non-breastfed children. Appropriate feeding frequency was coded as 1 (minimum meal frequency) and inappropriate was coded 0 (low meal frequency) [21].

Other potential predictors in this study included sex of the baby (male vs female), prematurity (normal vs premature babies), and birth weight (normal and low birth weight as <2.5kg), breastfeeding duration (continue breastfeeding in months), and mothers’ age in years (<24, 25–34, >35). Others include mothers’ education level (no education, primary, secondary and higher education level), marital status (single, married/cohabiting and divorced/separated), alcohol intake (yes, no), and HIV status (positive, negative). Others include religion (Christian, Muslim), employment status (formal and informal employment), gravidity (1, 2–4, >4), parity (0, 1–4, >4), socioeconomic status (low, medium and high socio-economic status), multiple births (singleton and twins), and the number of visits at the clinic and enrollment year.

### Statistical analysis

Data were extracted from women and infant questionnaires of the parent study using EpiInfo 7.1.5.2 database and the analysis were done in StataCorp version 14.0. The overall proportion of stunting, wasting, and underweight was calculated and further analysis was done across the follow-up visits to see the peak of undernutrition. The proportion of children with suboptimal complementary feeding practices across the cohort and at different age points during the follow-up visit was calculated. A chi-squared test was used to determine any statistical difference in the proportion of children with inappropriate feeding practices across the nutrition indicators.

Mother-child pairs were followed from birth with repeated measurements done at 6, 9, 12, 15, and 24-months visits (including anthropometric measurements, food groups consumed, and feeding frequency). Some mothers had more than one pregnancy in the cohort hence there was a possible similarity among children of the same mother than those of different mothers. Therefore, to account for the correlation of repeated measures within a child and clustering effect of mothers and their children, multilevel modeling using “gllamm” a Stata command specifying cluster variable (mother-child), family (binomial), and link function (logit) were used. Mothers were cluster level three, each child was a cluster in level two, and repeated measures done in a child was a level one cluster.

Data were transformed into a long format. An empty model with no exposure variable was done to determine the total variability in stunting, wasting, and underweight that can be explained by clusters (ICC). To test the significance of each level of a cluster, the likelihood ratio test was used by comparing a model without a cluster versus a model with a cluster level. A nested model was considered important at the significant level of 5 percent. Univariable multilevel logistic regression analysis was done for factors in each cluster’s level (from level one to three) and factors that remained to be significant at a p-value of <10% were retained for a multivariable model. Fixed and random effects were tested and the final model comprises of a combined model with level 1, 2 and 3 factors. Therefore, multilevel logistic regression analysis was used to estimate the adjusted relative risk (ARR) with their 95 percent confidence interval for the effect of inappropriate complementary feeding practices and nutritional status of the children.

### Ethical consideration

Ethical approval was obtained from the Research Ethical Committee of Kilimanjaro Christian Medical University College (CREC No. 2411). The permission to use Pasua and Majengo cohort data was obtained from the Better Health for African Mother and Child (BHAMC) project. Written consents were obtained from mothers and numbers were used to identify participants instead of names to ensure confidentiality. All participants’ information was kept and analyzed anonymously.

## Results

### Baseline characteristics

About 3355 children aged 6–24 months were included in the analysis. The overall follow-up rate was 85 percent and follow-up rates at visit months 6, 9, 12, 15, and 24 were 86, 87, 88, 88, and 66 percent respectively. Some children were lost due to the absence of the mother at the clinic during the follow-up visit.

Total mothers in this analysis were 2774, each mother had 1 to 6 children, and each child had repeated observations ranging from 1 to 7 occasions/visits. The mean age of mothers at recruitment was 25.6 (SD ± 5.01) years and their partners’ age was 31.7 (SD ± 6.3) years. Most mothers (89.5%) were either married or cohabiting and 74.8 percent had primary level education. The majority (81.8%) had only one pregnancy in the cohort while others had up to six pregnancies. Regarding the children’s characteristics, 57 percent of the babies born in the cohort were males and the mean birth weight was 3.2 (SD ± 0.49) (Table 1).

**Table 1:** Child and maternal baseline characteristics (N = 3355).

Variables	Frequency	Percent
**Sex of the child**		
Male	1,922	57.3
Female	1,433	42.7
**Premature or term**		
Term	3,308	98.6
premature	47	1.4
**Singleton or twin**		
Singleton	3,316	98.8
Twin	39	1.2
**Age of the mother**		
<24	1,408	42.0
25–34	1,759	52.4
>35	188	5.6
**Education Level**		
None	103	3.1
Primary	2,509	74.8
Secondary and above	743	22.2
**Employment Status**		
Informal employment	2,407	71.7
Formal employment	948	28.3
**Marital status**		
Married/cohabiting	3,002	89.5
Single	221	6.6
Widowed/divorced/separated	132	3.9
**Gravidity**		
Gravid 1	1,291	38.5
Gravid 2–4	1,824	54.4
More than 4	240	7.1
**Parity**		
0	1,291	38.5
1–4	1,951	58.2
Above 5	113	3.4
**Alcohol intake**		
No	2,318	69.1
Yes	1,037	30.9
**Mode of Delivery**		
Vaginal Delivery	3,025	90.2
Caesarean section	330	9.8
**HIV Status**		
Negative	2,893	86.2
Positive	462	13.7
**Age of the partner**		
< = 24	457	13.6
>24	2,898	86.4
**Education of the partner**		
None	28	0.8
Primary	2,661	79.3
Secondary and above	666	19.9
Socio-economic status* **(N = 1436)**		
Low	545	38.0
Medium	549	38.2
High	342	23.8

* Variable with missing information

### Nutrition status of children aged 6–24 months

**Fig 2:**
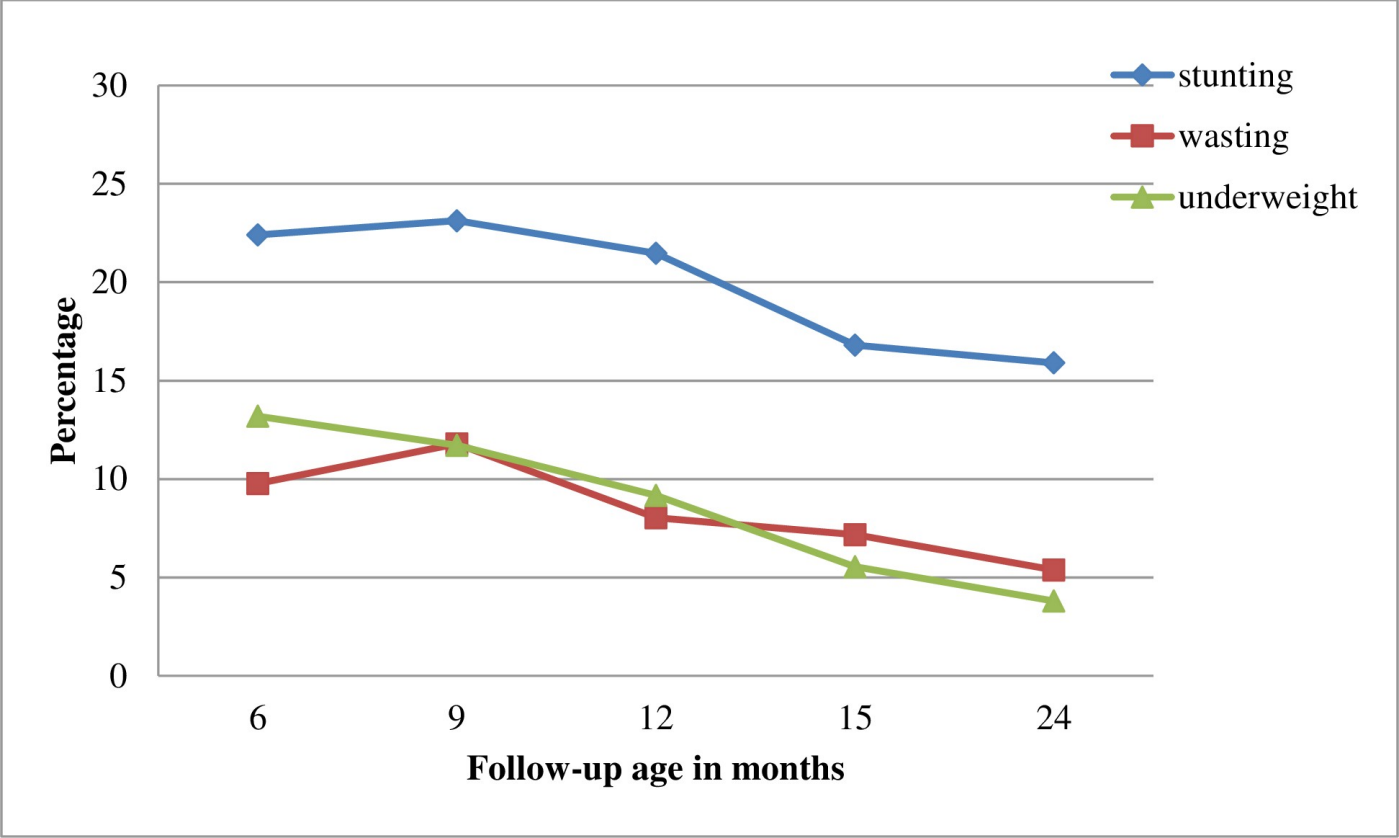
Proportion of children with undernutrition across follow up months.

The overall proportion of stunting, wasting, and underweight was 20.7, 8.9, and 9.7 respectively. All three indicators of undernutrition started declining from six to 24 months of age. Stunting was the most prevalent condition but a high proportion of children who were stunted, underweight, and wasted were observed within the first nine months of life (Fig 2).

### Complementary feeding practices

**Fig 3:**
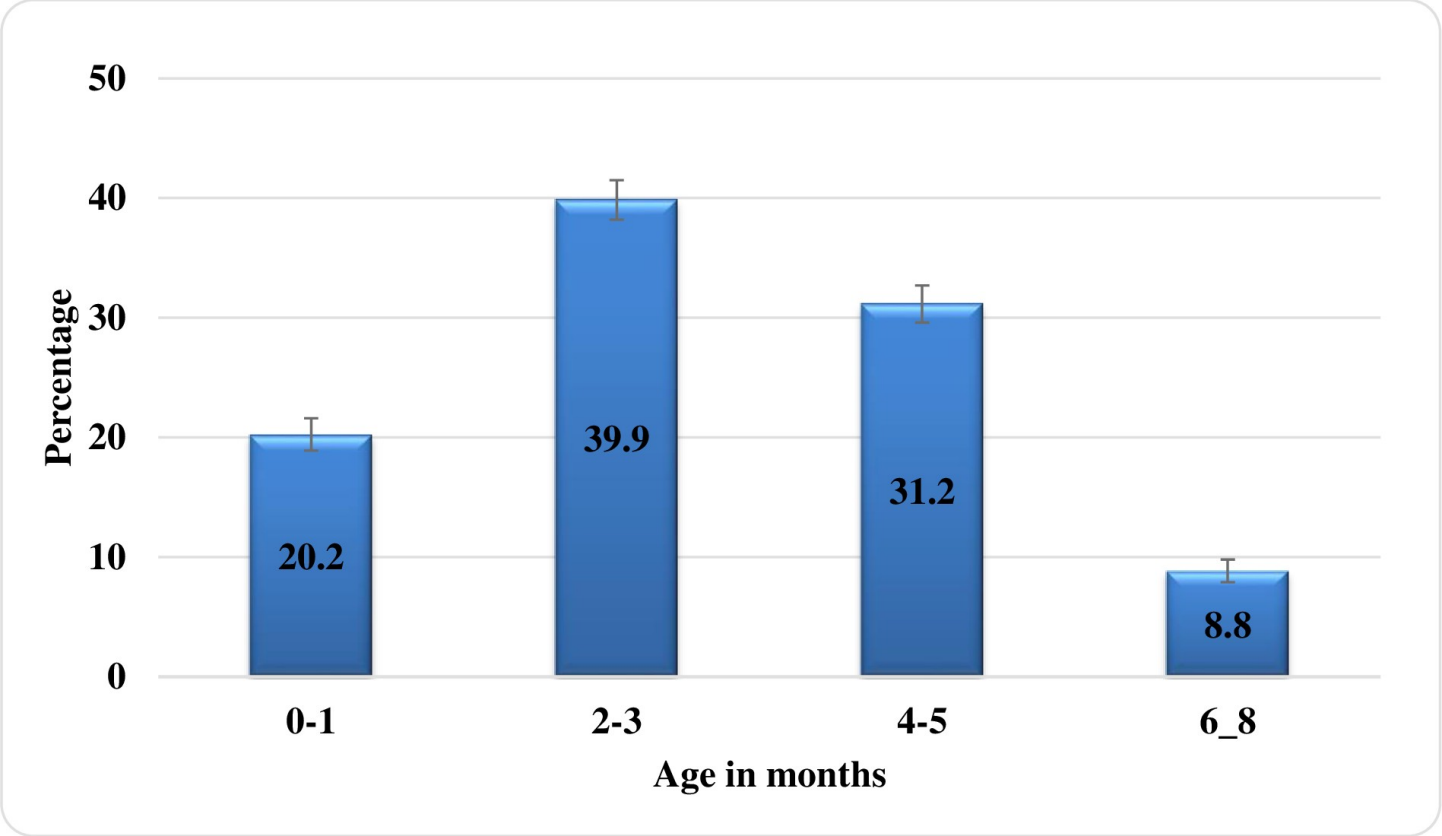
Distribution of children according to age at introduction of complementary feeding.

**Fig 4:**
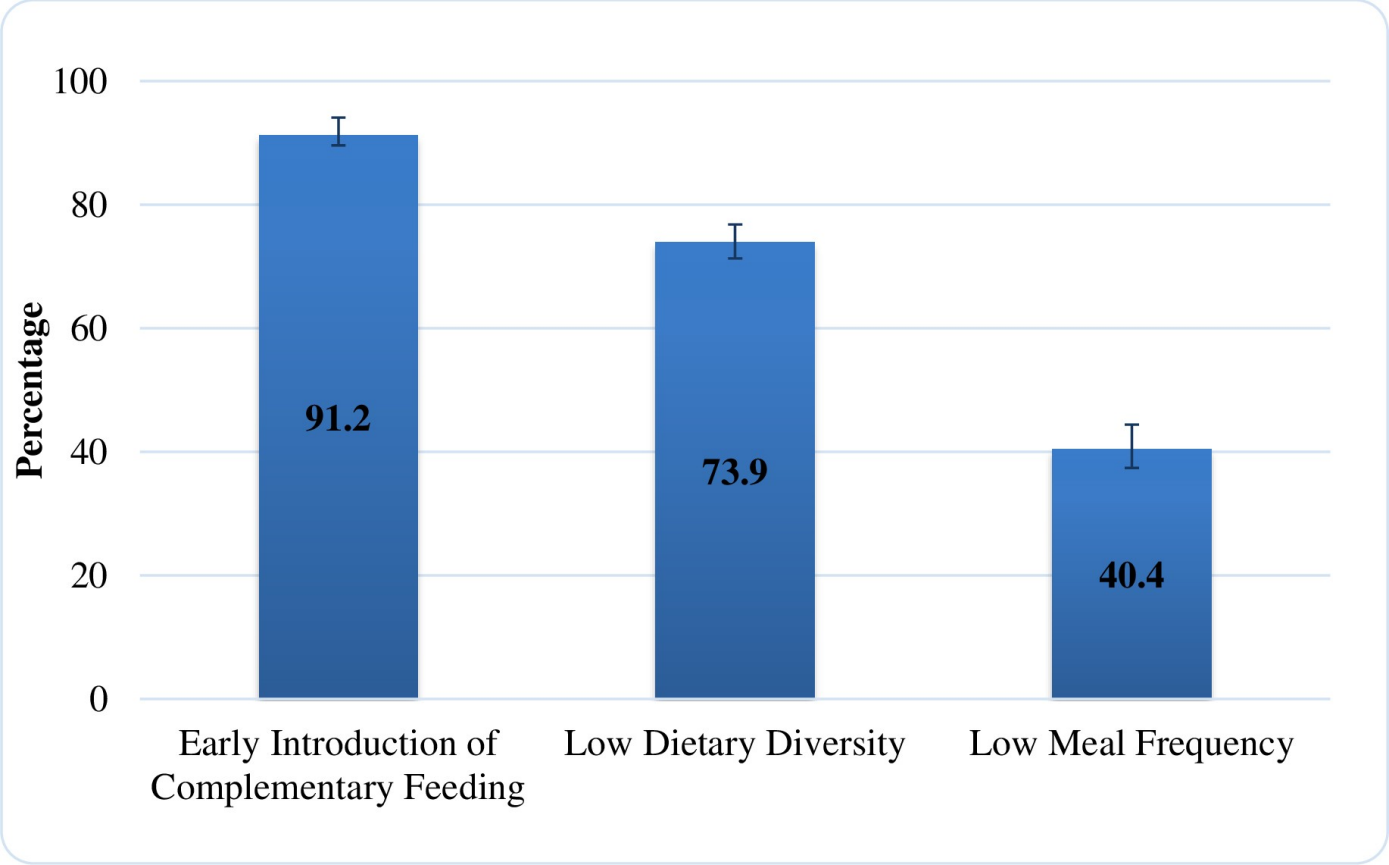
Proportion of inappropriate complementary feeding practices among children aged 6–24 months.

The mean age of introduction of complementary food was 3 months. The majority of children (39.9%) were initiated complementary food between 2 to 3 months of age, whereby only 8.8 percent reached the recommended age of 6 months (Fig 3). The majority of children (91.2%) were introduced to complementary food before six months, the majority of children (73.9%) had low dietary diversity, and 40.4 percent had low meal frequency (Fig 4).

**Fig 5:**
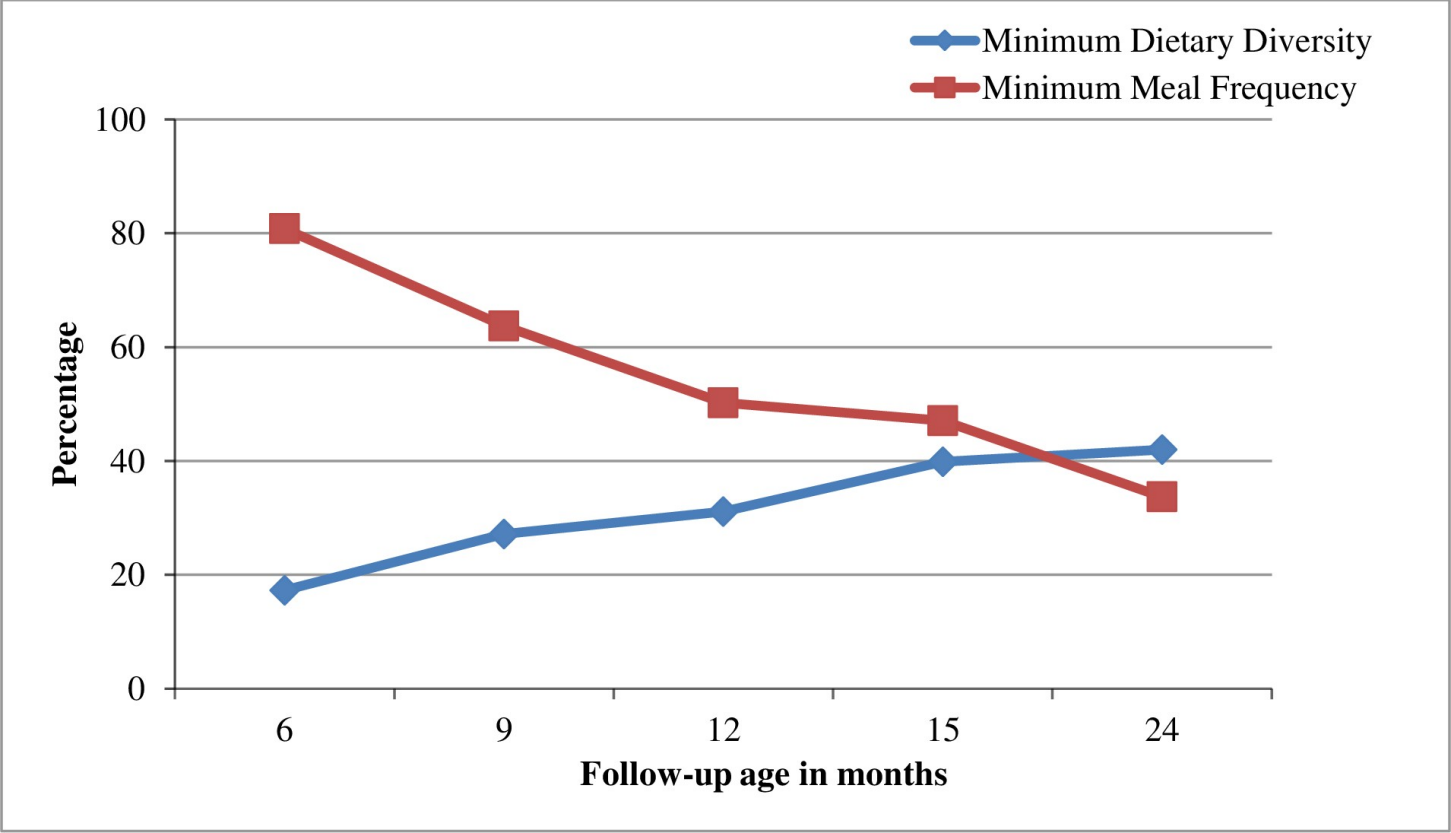
Proportion of children who adhered to the minimum meal frequency and minimum dietary diversity across follow up visits.

The proportions of children who adhered to the minimum dietary diversity slightly increased across follow-up months. While on the other hand, the proportions of children who adhered to the minimum meal frequency decreased across the follow-up visit (Fig 5).

**Fig 6:**
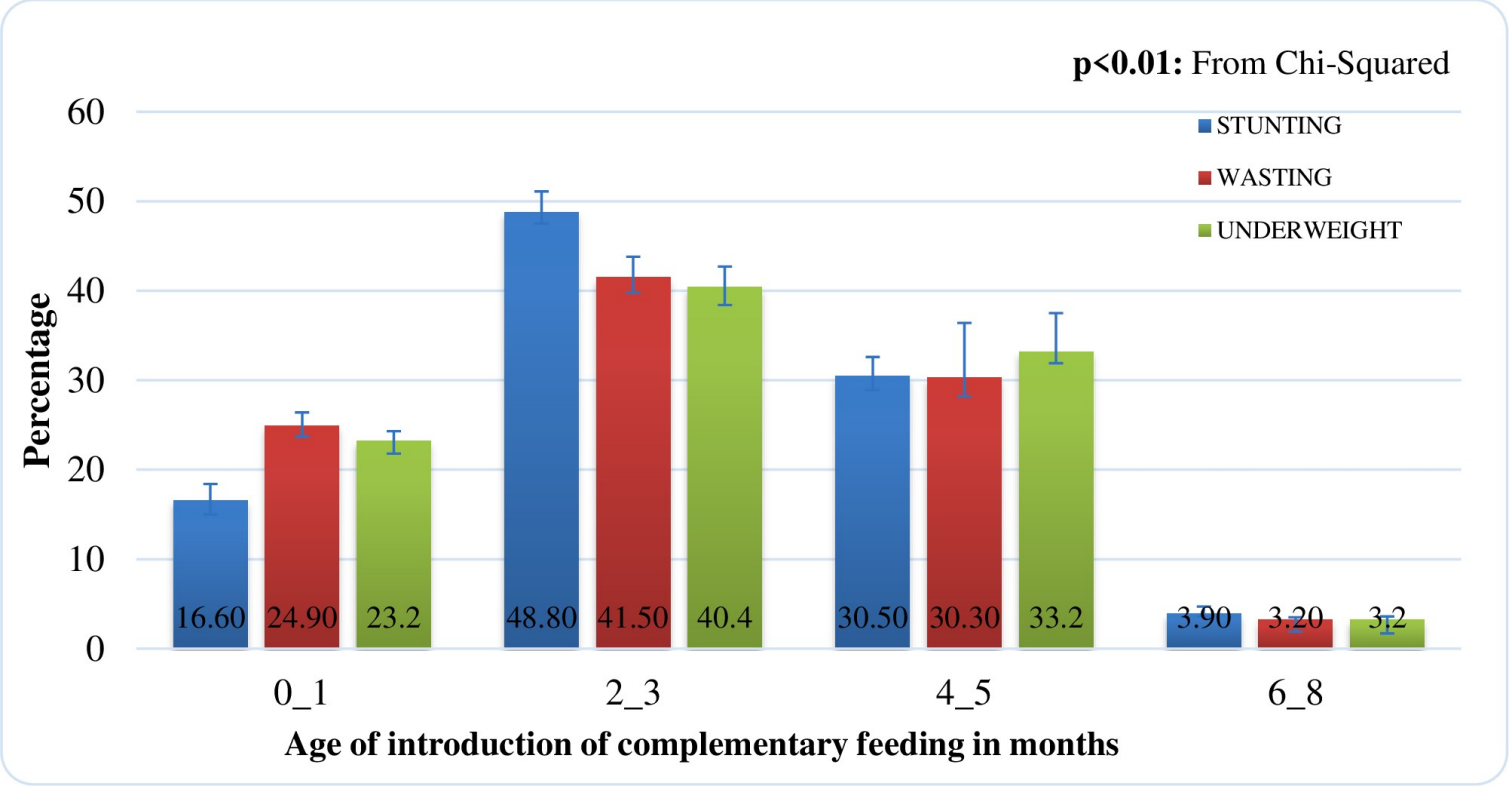
Proportion of children with undernutrition with respect to age category at which complementary feeding was introduced.

The highest proportion of children, who were stunted, wasted, and underweight was observed among children who were introduced to complementary feeding before the recommended age of 6 months. This shows a significant difference in the proportion of children who were stunted, wasted, and underweight across age categories at which complementary feeding was introduced with p-value <0.01 (Fig 6).

### Multilevel regression analysis for the effect of inappropriate complementary feeding practices on nutritional status of the children

After adjusting for the child and maternal characteristics, infants that were introduced to complementary foods at age 2–3 months had approximately 2 times higher risk of being stunted (ARR 1.88, 95%CI 1.06–3.36, p = 0.032). Infants that were introduced to complementary foods at age 0–1 month had 2.9 and 2.6 times higher risk of being wasted and underweight as compared to those who were introduced to complementary foods between 6–8 months (ARR 2.86, 95% CI: 1.30–6.29, p = 0.009 and ARR 2.57 95%CI: 1.29–5.14, P = 0.007).

Children who did not receive the minimum meal diversity had 29 percent higher risk of stunting compared with children who received the minimum meal diversity (ARR 1.29, 95%CI 1.01–1.64, p = 0.041). Children who did not receive the minimum meal frequency had approximately 3 times higher risk of stunting (ARR 2.87, 95%CI 2.30–3.59, p < 0.001). Infants who did not receive the minimum meal frequency had 93 and 89 percent higher risk of wasting and underweight, respectively, as compared with children who received the minimum meal frequency (ARR 1.93 95% CI 1.49–2.46, p<0.001 and ARR 1.89; 95% CI 1.52–2.35, p < 0.001) (Table 2).

**Table 2:** Multilevel logistic regression: Effect of inappropriate complementary feeding practices on the nutrition status of the children.

Variables	Stunting		Wasting		Underweight	
	CRR (95% CI)	ARR(95% CI)	CRR (95% CI)	ARR(95% CI)	CRR (95% CI)	ARR(95% CI)
**Age of introduction of Complementary Feeding**						
6–8 months	1	1	1	1	1	1
0–1 months	0.89 (0.64–1.24)	1.41 (0.77–2.16)	1.75 (1.07–2.84)*	2.86 (1.30–6.29)**	1.68(0.97–2.93)	2.57 (1.29–5.14)**
2–3 months	1.40 (1.03–1.91)*	1.88 (1.06–3.36)*	1.29 (0.81–2.06)	1.75 (0.81–3.77)	1.64(0.81–2.36)	1.68 (0.85–3.32)
4–5 months	1.30 (0.95–1.78)	1.42 (0.78–2.59)	1.43 (0.89–2.30)	1.95 (0.89–4.28)	1.93(1.09–3.21)*	2.14 (1.08–4.29)*
**Minimum Dietary Diversity**						
MDD	1		1	1	1	1
Low MDD	1.02 (0.89–1.16)	1.29 (1.01–1.64)*	1.05 (0.87–1.27)	1.19 (0.89–1.57)	1.17 (0.95–1.43)	1.13 (0.89–1.44)
**Minimum Meal Frequency**						
MMF	1		1	1	1	1
Low MMF	3.15 (2.80–3.54)***	2.87 (2.30–3.59)***	1.37 (1.17–1.60)***	1.93 (1.49–2.49)***	1.38 (1.18–1.63)***	1.89 (1.52–2.35)***

CRR = Crude Risk Ratio

ARR = Adjusted Risk Ratio; Risk Ratio Adjusted for sex of the baby, premature and birth weight, breastfeeding duration, mothers’ age, mother’s education level, marital status, alcohol intake, HIV status, religion, number of visits and enrollment year.

* Significant at p<0.05

** Significant at p<0.01

*** Significant at p<0.001

## Discussion

The result of this study showed that suboptimal complementary feeding practices were common in urban Moshi. Early introduction of complementary feeding before 6 months was associated with a higher risk of stunting, wasting, and underweight. Children aged 6–24 months with low minimum meal frequency were more likely to be stunted, wasted, and underweight. Children with low MDD were likely to be stunted.

Our findings show that about 91 percent of the children were given complementary food before the age of 6 months. This was higher than the national prevalence of 41 percent from the 2015/16 Tanzania Demographic Health Survey and was slightly higher for Kilimanjaro region of 85.4 percent [8]. The differences in the study design could be the reason for the observed differences since the TDHS is a cross sectional study and only one data point is collected, whereas this was a cohort study with minimum recall bias. Similar results have been reported in a study carried out in Kenya which found 98 percent of infants were complemented before the age of 6 months [24]. The main reason for this high proportion of inappropriate age of introducing complementary food could be the mothers’ perception that breast milk is insufficient for a child’s growth as reported in a study carried out in Tanga, northern Tanzania [25]. Therefore, health education to mothers is essential in promoting appropriate age of introduction of complementary feeding especially during the ante and post-natal period [26].

Our findings show the prevalence of, 26 percent of low MDD, which is similar to the national level [8]. It is common in this setting for the infant to receive porridge, *“mtori*,*”* or *“kitawa”* when they reach six months of age as it is perceived to be the food for infants and that infants cannot eat other foods such as vegetables and animal products. It has been reported that poor dietary diversity is a common practice in SSA countries and starch-based foods such as porridge is the commonly given food [27]. A study in Nigeria, Zambia, and Ethiopia also reported a low rate of dietary diversity [28, 29]. In Nigeria, inadequate maternal nutrition knowledge on child feeding or economic reasons were cited as among the contributing factors [29].

In our study, MMF was observed to be about 60 percent, which was higher than the reported 39 percent at the national level [8]. A comparable result was reported in Zambia and Ethiopia [28]. The reason for this could be food preparation, as reported earlier that the common food given is porridge, *“mtori*,*”* or *“kitawa*.*”* It is common to prepare these foods mostly in the morning and put it in a flask where a mother or another family member could feed the infant throughout the day hence receiving the required number of meals in a day.

Our findings indicated that early introduction of complementary feeding was significantly associated with a higher risk of stunting, wasting, and underweight. Studies done in Zambia, Ghana, and Nigeria reported similar findings [28–30]. Early introduction of complementary feeding is linked with a substantially increased risk of recurrent diarrhoea and other infectious diseases resulting in undernutrition [31, 32]. In LMICs, access to clean and safe water is a challenge leading to microbial contamination of complementary foods through unsafe preparation and storage. On the other hand, the gastrin intestinal track of an infant below six months of age is not fully developed to digest other foods apart from breast milk [33–36]. Therefore, there is a need of continuing providing education on the advantages of exclusive breastfeeding to mothers/caregivers. This can be done during antenatal care, delivery, postnatal visits, and monthly child growth monitoring visits.

Our findings also showed that children who received low minimum meal frequency were more likely to be stunted, wasted, and underweight than is the case with their peers. Our findings were similar to the findings of other studies in most low and middle-income countries [11, 29]. The minimum number of meals per day is required in order to attain the necessary level of energy and nutritional requirement and prevent deficiencies that could result into undernutrition [37]. These findings are in contrast with the findings in a study done in Ghana which found that minimum meal frequency was not associated with stunting [30]. The apparent lack of association in this study may be because there was very little variation in the study population with respect to this indicator.

Low MDD was associated with stunting and not wasting or underweight. Our findings were in line with the findings in other studies that used a variety of indicators aimed at capturing food variety or dietary diversity in most low and middle-income countries [11, 28, 29, 38–40]. This is because a more diversified diet is highly correlated with adequate energy and protein, micronutrients, and animal source food [41, 42]. The inadequate intake of a quality diet is among the main contributing factors for undernutrition [43]. We also anticipate a significant contribution of maternal nutrition to children’s diet and nutritional status. However, the parent study did not capture variables that assessed the nutritional status of women and dietary habits during pregnancy. Hence, we recommend future studies to establish the contribution of maternal nutrition to the infant’s diet and nutritional status.

### Strength and limitation of the study

The strength of this study was that this was the first study to examine the effect of inappropriate complementary feeding practices on the child’s nutritional status in Tanzania. The close follow-up of mother-child pairs provide more accurate estimates of the effect of complementary feeding practices on the child’s nutritional status. Hierarchical modelling of the data provided more reliable estimates as it accounts for the correlation of the repeated measurements and the effect of clustering [44, 45].

Despite the strength of this study, some limitations had to be taken into account while interpreting the findings. First, there was a loss to follow-up bias of about 14.3 percent. Therefore, the information of those who were lost was not analyzed. However, this rate was low compared to the recommended threshold level [46]. In addition, we missed some variables such as those of assessing maternal health during pregnancy (as parent study recruited women in their third trimester), which could have the potential effect on children’s diet and nutritional status. Therefore, the significant contribution of maternal nutrition was not assessed.

### Conclusion and recommendation

We observed a high proportion of children with suboptimal complementary feeding practices. Early introduction of complementary food increased the risk of stunting, wasting, and underweight. Children who had low meal frequency were more likely to be stunted, wasted, and underweight than was the case with their counterparts. Children who had low dietary diversity were more likely to be stunted.

There is a need for Tanzania’s Ministry of Health and other stakeholders to formulate strategies for educating mothers both in the health facility and in the community on the importance of adhering to the recommended age of introduction of complementary feeding, meal frequency, and dietary diversity. Also, health care providers should be informed on the low proportion of adherence to the recommended complementary feeding practices so that they can discuss with mothers/caregivers the importance of optimal complementary feeding practices during ANC, delivery, postnatal visit and also during child growth monitoring and vaccination visits. Future studies are recommended to assess women’s knowledge and understanding of optimal complementary feeding practices so as to know the reasons for the observed high proportion of children with suboptimal complementary feeding. In addition, other studies should establish the contribution of maternal nutrition on infants’ diet and nutritional status.

## Supporting information

S1 Appendix Additional tables for multilevel logistic regression for the effect of inappropriate complementary feeding practices on the nutritional status of the children aged 6–24 months. (DOCX)

S1 File A copy of questionnaire. (PDF)
