# Supplementary material for: Effect of inappropriate complementary feeding practices on the nutritional status of children aged 6-24 months in urban Moshi, Northern Tanzania: Cohort study
Source: PLoS One. 2021 May 13;16(5):e0250562. doi: 10.1371/journal.pone.0250562 (PMC8118559; doi:10.1371/journal.pone.0250562)
Supplement: S1 Appendix — (DOCX) [file pone.0250562.s001.docx]

## S1 Appendix: Multilevel Logistic Regression model.

Table 1: Effect of inappropriate complementary feeding practices on stunting

| **Variables** | CRR | 95% CI | p-value | ARR | 95% CI | p-value |
| --- | --- | --- | --- | --- | --- | --- |
| **Age of Complementary Feeding** |  |  |  |  |  |  |
| 6-8 months | 1 |  |  | 1 |  |  |
| 0-1 months | 0.89 | 0.64 – 1.24 | 0.500 | 1.41 | 0.77 - 2.16 | 0.267 |
| 2-3 months | 1.40 | 1.03 – 1.91 | 0.031 | 1.88 | 1.06 – 3.36 | 0.032 |
| 4-5 months | 1.30 | 0.95 – 1.78 | 0.105 | 1.42 | 0.78 – 2.59 | 0.250 |
| **Minimum Dietary Diversity** |  |  |  |  |  |  |
| Yes | 1 |  |  | 1 |  |  |
| No | 1.02 | 0.89 – 1.16 | 0.774 | 1.29 | 1.01 – 1.64 | 0.041 |
| **Minimum Meal Frequency** |  |  |  |  |  |  |
| Yes | 1 |  |  | 1 |  |  |
| No | 3.15 | 2.80 – 3.54 | <0.001 | 2.87 | 2.30 – 3.59 | <0.001 |
| **Breastfeeding duration** | 0.995 | 0.993–0.997 | <0.001 | 0.998 | 0.995 – 1.0 | 0.189 |
| **Age of the mother** |  |  |  |  |  |  |
| <24 | 1 |  |  | 1 |  |  |
| 25-34 | 1.24 | 1.09 – 1.42 | <0.001 | 0.86 | 0.68 - 1.09 | 0.211 |
| >35 | 1.07 | 0.80 – 1.44 | 0.653 | 0.91 | 0.58 - 1.44 | 0.699 |
| **Mother’s Education Level** |  |  |  |  |  |  |
| Primary | 1 |  |  | 1 |  |  |
| Secondary and above | 0.63 | 0.52 – 0.76 | <0.001 | 0.88 | 0.61 – 1.26 | 0.481 |
| None | 1.15 | 0.81 – 1.63 | 0.430 | 1.85 | 1.16 – 2.95 | 0.009 |
| **Marital status** |  |  |  |  |  |  |
| Single | 1 |  |  | 1 |  |  |
| Married | 0.77 | 0.58 – 1.02 | 0.065 | 0.89 | 0.57 - 1.38 | 0.592 |
| Widowed/divorced/separated | 1.37 | 1.01 – 1.85 | 0.043 | 1.44 | 0.62 – 3.30 | 0.396 |
| **HIV Status** |  |  |  |  |  |  |
| Negative | 1 |  |  | 1 |  |  |
| Positive | 1.35 | 1.13 – 1.62 | 0.001 | 0.92 | 0.61 – 1.40 | 0.694 |
| **Religion** |  |  |  |  |  |  |
| Christian | 1 |  |  | 1 |  |  |
| Muslims | 0.88 | 0.77 – 0.99 | 0.049 | 1.02 | 0.81 – 1.29 | 0.847 |
| **Alcohol drinking** |  |  |  |  |  |  |
| No |  |  |  | 1 |  |  |
| Yes | 0.60 | 0.52 – 0.70 | <0.001 | 0.93 | 0.72 – 1.23 | 0.647 |
| **Sex of the child** |  |  |  |  |  |  |
| Male | 1 |  |  | 1 |  |  |
| Female | 0.39 | 0.34 – 0.44 | <0.001 | 0.38 | 0.31 - 0.48 | <0.001 |
| **Premature or term** |  |  |  |  |  |  |
| Premature | 1 |  |  | 1 |  |  |
| Term | 2.16 | 1.25 – 3.72 | 0.006 | 1.009 | 0.47 – 2.16 | 0.982 |
| **Birthweight** |  |  |  |  |  |  |
| Normal >2.5 | 1 |  |  | 1 |  |  |
| Low <2.5 | 3.39 | 2.73 – 4.19 | <0.001 | 3.82 | 2.57 – 5.68 | <0.001 |
| **Diseases** |  |  |  |  |  |  |
| Yes | 1 |  |  | 1 |  |  |
| No | 0.89 | 0.78 – 1.02 | 0.094 | 0.96 | 0.77 – 1.18 | 0.666 |
| **Enrolment year** |  |  |  |  |  |  |
| 2002/2005 | 1 |  |  | 1 |  |  |
| 2006/2013 | 1.65 | 1.41 – 1.93 | <0.001 | 2.08 | 1.31 – 3.29 | 0.002 |
| 2014/2015 | 0.50 | 0.37 – 0.67 | <0.001 | 1.30 | 0.57 – 2.97 | 0.528 |
| **Employment status** |  |  |  |  |  |  |
| Formal employment | 1 |  |  |  |  |  |
| Informal employment | 1.21 | 1.03 – 1.41 | 0.019 |  |  |  |
| **Visits** | 0.97 | 0.96 – 0.98 | <0.001 | 0.96 | 0.92 – 1.00 | 0.069 |

Table 2: Effect of inappropriate complementary feeding practices on Wasting

| **Variables** | CRR | 95% CI | p-value | ARR | 95% CI | p-value |
| --- | --- | --- | --- | --- | --- | --- |
| **Age of Complementary Feeding** |  |  |  |  |  |  |
| 6-8 months | 1 |  |  | 1 |  |  |
| 0-1 months | 1.75 | 1.07 – 2.84 | 0.025 | 2.86 | 1.30 - 6.29 | 0.009 |
| 2-3 months | 1.29 | 0.81 – 2.06 | 0.290 | 1.75 | 0.81 - 3.77 | 0.155 |
| 4-5 months | 1.43 | 0.89 – 2.30 | 0.144 | 1.95 | 0.89 - 4.28 | 0.097 |
| **Minimum Dietary Diversity** |  |  |  |  |  |  |
| No | 1 |  |  | 1 |  |  |
| Yes | 1.05 | 0.87 – 1.27 | 0.619 | 1.19 | 0.89 – 1.57 | 0.237 |
| **Minimum Meal Frequency** |  |  |  |  |  |  |
| No | 1 |  |  | 1 |  |  |
| Yes | 1.37 | 1.17 – 1.60 | <0.001 | 1.93 | 1.49 – 2.49 | <0.001 |
| **Breastfeeding duration** | 0.995 | 0.99 – 0.997 | <0.001 | 0.999 | 0.996 – 1.00 | 0.790 |
| **Age of the mother** |  |  |  |  |  |  |
| <24 | 1 |  |  | 1 |  |  |
| 25-34 | 1.32 | 1.10 – 1.58 | 0.003 | 1.14 | 0.86 – 1.50 | 0.356 |
| >35 | 1.25 | 0.84 – 1.87 | 0.279 | 1.19 | 0.71 – 2.02 | 0.507 |
| **Mother’s Education Level** |  |  |  |  |  |  |
| Primary | 1 |  |  | 1 |  |  |
| Secondary and above | 1.05 | 0.83 – 1.34 | 0.686 | 0.98 | 0.64 – 1.50 | 0.928 |
| None | 1.51 | 0.95 – 2.39 | 0.078 | 1.77 | 1.04 – 3.04 | 0.037 |
| **HIV results** |  |  |  |  |  |  |
| Negative | 1 |  |  | 1 |  |  |
| Positive | 1.47 | 1.16 – 1.88 | 0.002 | 1.43 | 0.90 – 2.29 | 0.133 |
| **Employment Status** |  |  |  |  |  |  |
| Informal employment | 1 |  |  | 1 |  |  |
| Formal employment | 1.69 | 1.38 – 2.07 | <0.001 | 1.05 | 0.64 – 1.70 | 0.853 |
| **Sex of the child** |  |  |  |  |  |  |
| Male | 1 |  |  | 1 |  |  |
| Female | 0.43 | 0.36 – 0.52 | <0.001 | 0.63 | 0.48 - 0.81 | <0.001 |
| **Birthweight** |  |  |  |  |  |  |
| Normal >2.5 | 1 |  |  | 1 |  |  |
| Low <2.5 | 1.57 | 1.15 – 2.15 | 0.005 | 1.21 | 0.76 – 1.93 | 0.428 |
| **Enrolment year** |  |  |  |  |  |  |
| 2002/2005 | 1 |  |  | 1 |  |  |
| 2006/2013 | 1.73 | 1.41 – 2.13 | <0.001 | 1.48 | 0.86 – 2.56 | 0.159 |
| 2014/2015 | 1.18 | 0.83 – 1.67 | 0.360 | 1.28 | 0.49 – 3.33 | 0.610 |
| **Visits** | 0.95 | 0.94 – 0.97 | <0.001 | 0.92 | 0.90 – 0.95 | <0.001 |

Table 3: Effect of inappropriate complementary feeding practices on Underweight

| **Variables** | CRR | 95% CI | p-value | ARR | 95% CI | p-value |
| --- | --- | --- | --- | --- | --- | --- |
| **Age of Complementary Feeding** |  |  |  |  |  |  |
| 6-8 months | 1 |  |  | 1 |  |  |
| 0-1 months | 1.68 | 0.97 – 2.93 | 0.066 | 2.57 | 1.29 – 5.14 | 0.007 |
| 2-3 months | 1.39 | 0.81 – 2.36 | 0.229 | 1.68 | 0.85 – 3.32 | 0.134 |
| 4-5 months | 1.87 | 1.09 – 3.21 | 0.023 | 2.14 | 1.08 – 4.29 | 0.030 |
| **Minimum Dietary Diversity** |  |  |  |  |  |  |
| Yes | 1 |  |  | 1 |  |  |
| No | 1.17 | 0.95 – 1.43 | 0.134 | 1.13 | 0.89 – 1.44 | 0.323 |
| **Minimum Meal Frequency** |  |  |  |  |  |  |
| Yes | 1 |  |  | 1 |  |  |
| No | 1.38 | 1.18 – 1.63 | <0.001 | 1.89 | 1.52 – 2.35 | <0.001 |
| **Breastfeeding duration** | 0.994 | 0.99–0.997 | <0.001 | 0.999 | 0.996 – 1.0 | 0.343 |
| **Mother’s Education Level** |  |  |  |  |  |  |
| Primary | 1 |  |  | 1 |  |  |
| Secondary and above | 0.68 | 0.51 – 0.91 | 0.009 | 0.89 | 0.61 – 1.30 | 0.548 |
| None | 2.30 | 1.28 – 3.82 | 0.001 | 1.99 | 1.31 – 3.03 | 0.001 |
| **Marital status** |  |  |  |  |  |  |
| Single | 1 |  |  | 1 |  |  |
| Married | 0.88 | 0.57 – 1.36 | 0.561 | 0.89 | 0.57 - 1.41 | 0.627 |
| Widowed/divorced/separated | 2.02 | 1.28 – 3.19 | 0.003 | 1.60 | 0.78 – 3.30 | 0.201 |
| **Alcohol intake** |  |  |  |  |  |  |
| No | 1 |  |  | 1 |  |  |
| Yes | 0.72 | 0.56 – 0.91 | 0.006 | 0.98 | 0.76 – 1.28 | 0.904 |
| **HIV Status** |  |  |  |  |  |  |
| Negative | 1 |  |  | 1 |  |  |
| Positive | 1.53 | 1.15 – 2.02 | 0.003 | 1.29 | 0.87 – 1.92 | 0.205 |
| **Employment Status** |  |  |  |  |  |  |
| Informal employment | 1 |  |  | 1 |  |  |
| Formal employment | 0.76 | 0.59 – 0.998 | 0.048 | 0.95 | 0.63 – 1.45 | 0.821 |
| **Sex of the child** |  |  |  |  |  |  |
| Male | 1 |  |  | 1 |  |  |
| Female | 0.22 | 0.17 – 0.28 | <0.001 | 0.46 | 0.37 - 0.59 | <0.001 |
| **Birthweight** |  |  |  |  |  |  |
| Normal >2.5 | 1 |  |  | 1 |  |  |
| Low <2.5 | 3.14 | 2.23 – 4.42 | <0.001 | 1.93 | 1.37 – 2.71 | <0.001 |
| **Enrolment year** |  |  |  |  |  |  |
| 2002/2005 | 1 |  |  | 1 |  |  |
| 2006/2013 | 1.15 | 0.89 – 1.48 | 0.281 | 1.27 | 0.81 – 1.996 | 0.299 |
| 2014/2015 | 0.22 | 0.12 – 0.39 | <0.001 | 1.22 | 0.53 – 2.84 | 0.640 |
| **Number of visits** | 0.91 | 0.89 – 0.92 | <0.001 | 0.92 | 0.90 – 0.94 | <0.001 |
