# Supplementary material for: Effect of inappropriate complementary feeding practices on the nutritional status of children aged 6-24 months in urban Moshi, Northern Tanzania: Cohort study
Source: PLoS One. 2021 May 13;16(5):e0250562. doi: 10.1371/journal.pone.0250562 (PMC8118559; doi:10.1371/journal.pone.0250562)
Supplement: S1 File — (PDF) [file pone.0250562.s002.PDF]

FOMU YA MTOTO **MWEZI WA 56 (4 YEARS & 8 MONTHS)**

Tarehe____________________ **ID Number**__________ **Sex**_________

Date of birth ____________________ **Umri wa mtoto kwa miezi** ___________

JAZA UZITO WA MIEZI ILIYOONYESHWA HAPO CHINI KOPI GROWTH CARD

36M:_____ 40M:_____ 44M:_____ 48M:_____ 52M:_____

1.Je huyu ni mtoto wako wa ngapi? ____________________

Mkubwa wake aliyemuachia ziwa ana umri gani __________ Mdogo wake ana umri gani__________

2.Je mtoto ameshawahi kuugua tangu alipokuwa na umri wa miaka minne (48m) hadi sasa? 0.Hapana

1.Ndiyo..Tatizo na dawa alizopewa________________________________

...Aliugua kwa muda gani/siku ngapi? ________________________________

3.Je mtoto ameshalazwa hospitalini katika kipindi alipokuwa na miaka minne hadi sasa?)

0.Hapana 1.Ndiyo (*Tatizo* ________________________________)

4.Je mwanao ameshawahi kupewa dawa ya minyoo tangu alipokuwa na umri wa miaka minne?

0.Hapana 1.Ndiyo (Mara ngapi hadi sasa?____________________

5.Kuhusiana na chakula cha mtoto……..

Je anakula mwenyewe au analishwa? 0.Anakula mwenyewe 1.Analishwa

Je anapakuliwa sahani yake mwenyewe au mnakula pamoja 0.Yake mwenyewe 1.Pamoja

Je bado unampikia chakula chake mwenyewe tofauti na cha watu wazima?

0.Hapana 1.Ndiyo, kipi________________________________

Je kwa kawaida anakula mara ngapi kwa siku tangu anapoamka asubuhi hadi usiku? ______________

6.Na katika **masaa 24 yaliyopita au jana,** ni vyakula na vinywaji gani mtoto alipewa kuanzia alipoamka asubuhi hadi wakati wa kulala usiku. (*Andika kila kitu anachokitaja*). **(Last 24 – hours recall)**

Asubuhi: ____

____

Saa tatu – saa sita: ____

____

Mchana: ____

____

Saa nane – saa 12 jioni: ______________________________________

____

Jioni/Usiku: ____

7.Kama anampa uji uliochanganywa (ktk swali namba 6 au 8) taja vitu alivyochanganya____________

____

8.Je unampa mtoto samaki au dagaa? 0.Hapana 1.Ndiyo (Mara ngapi kwa wiki__________)

9.Maji ya kunywa mnachota wapi?

1.Bombani   2.Kisimani   3.Mtoni   4.Kwingine, taja________________

10.Unamchemshia mtoto maji ya kunywa? 0.Hapana   1.Ndiyo

11.Je mtoto ameshaweza kutumia choo mwenyewe au bado anatumia poti/ kujisaidia pembeni?

1.Anatumia poti   2.Anajisaidia mwenyewe chooni

12.Choo anachotumia mtoto ni   1.Cha shimo, hakijasakafiwa

2.Cha shimo, kimesakafiwa   3.Cha kuvuta   4.Vingine________________

13.Je mwanao anapenda kucheza?   0.Hapana   1.Ndiyo

Je ana watoto wa kucheza naye au yuko mwenyewe? 0.Anao   1.Yuko mwenyewe

Mara nyingi anacheza michezo gani? ________________

Anaweza kucheza kwa masaa mangapi kwa siku? ________________

Je anaweza kutembea mwenyewe kwenda kanisani/ msikitini?   0.Hapana   1.Ndiyo

Anapendelea kukaa na kuangalia TVau video kwa muda?   0.Hapana   1.Ndiyo 2.Hatuna TV

14. Je wakati anacheza na wenzie, anawaachie wachezee vitu vyake mfano mpira au mwanasesere au anawanyanganya?   0.Anawapa wachezee   1.Ni mchoyo, anawanyanganya

15.Je mtoto anaweza kufanya vifuatavyo peke yake?

Kuvaa viatu bila kuchanganya cha kushoto na kulia	0.Hapana	1.Ndiyo
Kula vizuri kwa kutumia kijiko	0.Hapana	1.Ndiyo
Kukimbia vizuri bila kudondoka dondoka	0.Hapana	1.Ndiyo
Kuruka kwa kutumia miguu yote (hopping)	0.Hapana	1.Ndiyo
Kusimama kwa mguu mmoja kwa sekunde kama 5	0.Hapana	1.Ndiyo
Ukirudi kukuambia kilichotokea mfano aliyempiga, kumyanganya kitu	0.Hapana	1.Ndiyo
Anaweza kukupigia hadithi za kitoto alizosikia shule/mtaani	0.Hapana	1.Ndiyo
Unaweza kumtuma akaleta ulichomtuma au jibu?	0.Hapana	1.Ndiyo
Anaweza kutaja watu anaoishi nao nyumbani	0.Hapana	1.Ndiyo
Kuhesabu hadi tano au zaidi	0.Hapana	1.Ndiyo
Anatambua ndugu aliowaona kwenye picha	0.Hapana	1.Ndiyo

17..Je nani anayefanya shughuli zifuatazo zinazomhusu mtoto wakati wa mchana?

a).Kutayarisha chakula cha mtoto ________________

b).Kuangalia mtoto anakula ipasavyo ________________

c).Kushinda na mtoto ________________

18.Je mtoto ameshaanza shule?   0.Hapana   1.Ndiyo

Kama bado unategemea kumuanzisha akiwa na umri gani? ________________

19.Akianza shule, je unaweza kuendelea kumleta ili apate huduma tukiwaambia muwalete siku ya jumamosi?   0.Hapana   1.Ndiyo

Kama ni hapana, tukimuhitaji katika muda unaotakiwa tutampataje?________________

Unataka kumuanzisha shule ipi? *Jina*________________

Je gharama yake kwa mwezi ni kiasi ghani?________________

FOMU YA MAMA MWEZI WA 56 (4 YRS & 8 MONTHS) BAADA YA KUJIFUNGUA

Date____________________ ID Number__________________________

Mwezi/Mwaka wa kuzaliwa____________________ Umri wa mama kwa miaka__________________

1.Je una mume/mwenzi unayeishi naye kwa sasa ? 0.Hapana 1.Ndiyo

Kama ndiyo umeishi naye kwa muda gani ? ______________________

2.Kwa hiyo hali yako ya unyumba kwa sasa ikoje?

1.Nimeolewa (Ni bwana au chuo cha ngapi? ______________________________)

2.Tunaishi pamoja bila ndoa (Ni bwana wa ngapi?__________________________)

3.Sijaolewa 4.Tumetengana (kwa miaka __________)/ Mjane (kwa miaka ____________)

3.Je bado unaishi na baba wa mtoto huyu aliye kwenye utafiti?

1.Ndiyo, tunaishi pamoja 2.Tumetengana (Mwaka ________baada ya kujifungua)

3.Hatujawahi kuishi pamoja

4.Je unatumia njia yoyoyte ya kujikinga usipate mimba?

0.Hapana 1.Ndiyo (Itaje/Zitaje______________________________)

5.Je mnatumia kondomu wakati mnapokutana kimwili na mwenzi/mume?

0.Hapana 1.Ndiyo (**Zungushia**; is it Occasional or Always?)

6.Je mpaka sasa mume au mwenzi unayeishi naye, umeshamwambia ulipima, na anajua majibu yako ya kipimo cha UKIMWI (HIV)? 0.Hapana 1.Ndiyo

7.Je na wewe unajua hali ya mwenza/mume kuhusiana na kipimo au majibu ua UKIMWI ? (**Know his serostatus?)** 0.Hapana 1.Ndiyo

8.Je mume/mwenzi wako naye ameshakuja kupima VVU (HIV)?

0.Hapana (**Kama bado, mshauri amlete**) 1.Ndiyo (**Walipeana majibu? 0 1)**

9.Kama mwenzi hajaja kupima/ au mama hajui status yake, je walishawahi kwenda sehemu nyingine inayotoa ushauri nasaha na kupima pamoja? (**couple counseled?**) 0.Hapana 1.Ndiyo

10.Kama mama hujui hali ya baba ya HIV, je anadhani anafanya mapenzi/ngono salama?

0.Hapana 1.Ndiyo 2.Nyinginezo, taja______________________________

11.Zaidi ya mwenzi umejadiliana majibu yako ya kipimo cha UKIMWI na mtu mwingine yeyote?

0.Hapana 1.Ndiyo (Mtaje/Wataje_____________________________)

12.Je kwa sasa una dalili zifuatazo?

1.Abnormal vaginal discharge	*Kutoka ute usio wa kawaida ukeni-unanuka, rangi njano n.k*	0	1
2.Genital itching	*Kuwashwa sehemu za siri*	0	1
3.Genital ulcers	*Vidonda sehemu za siri*	0	1
4.Dysuria	*Maumivu wakati wa kwenda kukujoa*	0	1
5.Dyspareunia	*Maumivu wakati wa kufanya mapenzi*	0	1
6.Lower abdominal pain	*Maumivu katika tumbo la uzazi*	0	1

INFANT EXAMINATION FORM : At 52 months (4 YRS and 4 MONTHS)

	No	Yes
1.General		
a. Looks pale at conjuctiva, palms	0	1
b. Hair changes	0	1
c. Oedema	0	1
d. Parotid enlargement	0	1
e. Scabies	0	1
2. Lymphadenopathy	**No**	**Yes**
a. Cervical	0	1
b. Axilla	0	1
3. Mouth	**No**	**Yes**
a. Oral thrush	0	1
b. Oropharyngeal ulcer	0	1
4.Respiratory system	**No**	**Yes**
a. Respiratory rate > 30/min	0	1
b. Any subcostal or intercostal recession	0	1
c. Any night sweats in the last 6 weeks	0	1
5. Gastro-intestinal Tract	**No**	**Yes**
a. Hepatomegaly	0	1
b. Splenomegaly	0	1
**6. Weight** ________ **Kg**		
**Height** ________ **Cm**		
**Head circumference** ________ **Cm**		
**7. a. Mid upper arm circumference (MUAC)** ________ **Cm**		
**8. b. Blood pressure (BP)** ________		

Pima HB (HemoCue machine) **→Kama ilikuwa chini ya 100g/L at 48 & 52 months**

→Kama hakupima at 48 months

Atoe choo kama hakupima at 48 months

Problems/ Diagnosis________________ Medication prescribed________________

Date of next visit 60M (5 years) ________________

3

17513

15713

Kama ana shida kati ya hizo mpe tiba syndromically kabla hata majibu ya HVS hayajaja

13.Je umeshalazwa hospitalini tangu ulipokuja kliniki kwa mara ya mwisho?

0.Hapana 1.Ndiyo (Tatizo________________________)

18.Je mama ana tatizo lingine lolote kuhusiana na afya yake? Litaje ________________________

19.Je kwa kawaida unakula mara ngapi kwa siku ukiweka kifungua kinywa/breakfast?______________

20. Je katika mwaka uliopita, umeshawahi kuchomwa sindano? 0.Hapana 1.Ndiyo

umeongezewa damu? 0.Hapana 1.Ndiyo

21.Je una shughuli ya kukuingizia kipato?

0.Sina shughuli 1.Shughuli, itaje ________________________)

Kipato kwa mwezi, akadirie____________________

Kama hapana, unamtegemea mwenzi kwa matumizi yote?______________________________

Kama hana shughuli, mshauri juu ya mikopo midogo inayotolewa na research aweze kujitegemea

KWA WALE AMBAO TAYARI NI POSITIVE

22.Je ameshapima kiasi cha CD4 katika miezi 6 iliyopita? 0.Hapana 1.Ndiyo (Kiasi__________)

(Kama hapana, mshauri afanye hivyo. Mpe referral kwenda Mawenzi iwapo CD4 ni 300mm^3 au chini.)

23.Je umeshaanza dawa za kurefusha maisha? (ARV's) 0.Hapana 1.Ndiyo, taja__________

HIV staging-WHO (jaza fomu stage aliyo leo) Stage 1 2 3 4 NA

PHYSICAL EXAMINATION

Palor-mucosa and conjuctiva 0.Normal 1.Pale

Oral thrush 0.Absent 1.Present

Lymphadenopathy (neck, axilla) 0.Absent 1.Present

Weight/Uzito (*kg*) ____________ **Height/Urefu (cm)**____________

Waist /Kiuno (cm) ____________ **Hip (cm)** ____________

Mid upper arm circumference________ **Blood Pressure**________________

→ Kama damu haikutolewa at 48 months, toa kwenye red na purple top na HVS

Problems/ Diagnosis ____________________ Management________________________

Tarehe ya next visit **60 months→(5 years)** ________________________

FOMU YA MTOTO **MWEZI WA 60 (5 YEARS)**

Tarehe____________________ **ID Number**__________ **Sex**_________

Date of birth __________________ **Umri wa mtoto kwa miezi** __________

JAZA UZITO WA MIEZI ILIYOONYESHWA HAPO CHINI KOPI GROWTH CARD

48M (4 years):________ 52M:__________ 56M:____________

1. Je huyu ni mtoto wako wa ngapi? __________________

   Mkubwa wake aliyemuachia ziwa ana umri gani ________ Mdogo wake ana umri gani___________

2. Je mtoto ameshawahi kuugua tangu alipokuwa na umri wa miaka minne (48m) hadi sasa? 0.Hapana

   1.Ndiyo..Tatizo na dawa alizopewa____

   ...Aliugua kwa muda gani/siku ngapi? ___________________________________

3. Je mtoto ameshalazwa hospitalini katika kipindi alipokuwa na miaka minne hadi sasa?)

   0.Hapana 1.Ndiyo (*Tatizo* ____________________________________)

4. Je mwanao ameshawahi kupewa dawa ya minyoo tangu alipokuwa na umri wa miaka minne?

   0.Hapana 1.Ndiyo (Mara ngapi hadi sasa?__________________

5. Kuhusiana na chakula cha mtoto……..

Je anakula mwenyewe au analishwa? 0.Anakula mwenyewe 1.Analishwa

Je anapakuliwa sahani yake mwenyewe au mnakula pamoja 0.Yake mwenyewe 1.Pamoja

Je bado unampikia chakula chake mwenyewe tofauti na cha watu wazima?

0.Hapana 1.Ndiyo, kipi_______________ _______________

Je kwa kawaida anakula mara ngapi kwa siku tangu anapoamka asubuhi hadi usiku?_____________

6. Je unampa mtoto samaki au dagaa? 0.Hapana 1.Ndiyo (Mara ngapi kwa wiki_________)

7. Unamchemshia mtoto maji ya kunywa? 0.Hapana 1.Ndiyo

8. Choo anachotumia mtoto ni 1.Cha shimo, hakijasakafiwa

   2.Cha shimo, kimesakafiwa 3.Cha kuvuta 4.Vingine______________

9. Je nani anayefanya shughuli zifuatazo zinazomhusu mtoto wakati wa mchana?

a).Kutayarisha chakula cha mtoto______________________________

b).Kuangalia mtoto anakula ipasavyo ______________________

c).Kushinda na mtoto ___________________________________

10. Je mtoto ameshaanza shule? 0.Hapana 1.Ndiyo

    Kama bado unategemea kumuanzisha akiwa na umri gani? ___________________________

11. Akianza shule, je unaweza kuendelea kumleta ili apate huduma tukiwaambia muwalete siku ya jumamosi? 0.Hapana 1.Ndiyo

    Kama ni hapana, tukimuhitaji katika muda unaotakiwa tutampataje?________________________

    Unataka kumuanzisha shule ipi? *Jina*___________ Gharama yake kwa mwezi?_____________

INFANT EXAMINATION FORM : At 52 months (4 YRS and 4 MONTHS)

1. General	No	Yes
a. Looks pale at conjuctiva, palms	0	1
b. Hair changes	0	1
c. Oedema	0	1
d. Parotid enlargement	0	1
e. Scabies	0	1

2. Lymphadenopathy	No	Yes
a. Cervical	0	1
b. Axilla	0	1

3. Mouth	No	Yes
a. Oral thrush	0	1
b. Oropharyngeal ulcer	0	1

4. Respiratory system	No	Yes
a. Respiratory rate > 20/min	0	1
b. Any subcostal or intercostal recession	0	1
c. Any night sweats in the last 6 weeks	0	1

5. Gastro-intestinal Tract	No	Yes
a. Hepatomegaly	0	1
b. Splenomegaly	0	1

6. **Weight** ________ **Kg**
Height ________ **Cm**

7. **Mid upper arm circumference (MUAC)** ________ **Cm**

8. **Blood pressure (BP)** ________

Laboratory

Pima Haemoglobin (HemoCue machine) ________

Problems/ Diagnosis________ Medication prescribed________

Date of next visit 66M (5 and half years) ________

FOMU YA MAMA **MWEZI WA 60 (<u>5 YEARS</u>) BAADA YA KUJIFUNGUA**

Date____________________ **ID Number** ____________________

Mwezi/Mwaka wa kuzaliwa________________ **Umri wa mama kwa miaka**______________

1.Je una mume/mwenzi unayeishi naye kwa sasa ? 0.Hapana 1.Ndiyo

Kama ndiyo umeishi naye kwa muda gani ? ____________________

2.Kwa hiyo hali yako ya unyumba kwa sasa ikoje?

1.Nimeolewa (Ni bwana wa ngapi/ au chuo cha ngapi? ____________________)

2.Tunaishi pamoja bila ndoa (Ni bwana wa ngapi? ____________________)

3.Sijaolewa

4.Tumetengana (kwa miaka ________) / Mjane (kwa miaka ____________)

3.Je bado unaishi na baba wa mtoto huyu aliye kwenye utafiti?

1.Ndiyo, tunaishi pamoja 2.Tumetengana (Mwaka ________baada ya kujifungua)

3.Hatujawahi kuishi pamoja

4.Je unatumia njia yoyoyte ya kujikinga usipate mimba?

0.Hapana 1.Ndiyo (Itaje/Zitaje____________________________)

5.Je mnatumia kondomu wakati mnapokutana kimwili na mwenzi/mume?

0.Hapana 1.Ndiyo (**Zungushia**; is it Occasional or Always?)

6.Je mpaka sasa mume au mwenzi unayeishi naye, umeshamwambia ulipima, na anajua majibu yako ya kipimo cha UKIMWI (HIV)? 0.Hapana 1.Ndiyo

7.Je na wewe unajua hali ya mwenza/mume kuhusiana na kipimo au majibu ua UKIMWI ? **(Know his serostatus?)** 0.Hapana 1.Ndiyo

8.Je mume/mwenzi wako naye ameshakuja kupima VVU (HIV)?

0.Hapana **(Kama bado, mshauri amlete)** 1.Ndiyo **(Walipeana majibu? 0 1)**

9.Kama mwenzi hajaja kupima/ au mama hajui status yake, je walishawahi kwenda sehemu nyingine inayotoa ushauri nasaha na kupima pamoja? **(couple counseled?)** 0.Hapana 1.Ndiyo

10.Je ulipata matatizo yoyote uliposhirikiana majibu yako ya HIV na mwenzi?

0.Hapana 1.Ndiyo (Yataje____)

2.Sijamshirikisha hadi sasa

11.Je kwa sasa una dalili zifuatazo?

1.Abnormal vaginal discharge	*Kutoka ute usio wa kawaida ukeni-unanuka, rangi njano n.k*	*0*	*1*
2.Genital itching	*Kuwashwa sehemu za siri*	0	1
3.Genital ulcers	*Vidonda sehemu za siri*	0	1
4.Dysuria	*Maumivu wakati wa kwenda kukujoa*	0	1
5.Dyspareunia	*Maumivu wakati wa kufanya mapenzi*	0	1
6.Lower abdominal pain	*Maumivu katika tumbo la uzazi*	0	1

CHILD BIRTH FORM **FOMU YA MTOTO**

NAMBA YA MTOTO **C ……………………………………………**

1. Date of delivery Tarehe ya kuzaliwa ……………………………………………

2. Are/ is the baby (ies). Je mtoto watoto ni 1. Single mmoja
   2. Twins Mapacha.

3. Is the baby Je mtoto wako ni 1. Premature (<37weeks) Njiti.
   2. Term (37 weeks >) Amekomaa.

4. Sex Jinsi ya Mtoto 1. Male Mme
   2. Female Mke

5. Weight uzito aliozaliwa nao. Kilo ………………….kg.

6. Length urefu aliozaliwa nao centimita …………cm.

7. Head circumference Mzunguko wa kichwa centimita……………cm.

8. Born Mtoto amezaliwa. 1. Alive Akiwa hai.
   2. Stillbirth Akiwa mfu.

9. APGAR score 1. After 1minute ……………………
   2. After 5 minutes ……………………

10. ARV given? Je mtoto amepewa ARV? 0. No Hapana.
    1. Yes Ndiyo.
    2. Not applicable, haihusiki.

    b. Date ARV given, tarehe aliyopewa ARV ………………………………………

    c. Type/s of ARV given, Aina ………………………………………

11. Opthalmia neonatorum present? Macho yana matongotongo 0. No Hapana
    1. Yes Ndiyo

12. Feeding option chosen, Je ni njia gani ya kumlisha mtoto aliyoichagua?
    1. EBF, kumnyonyesha bila kuchanganya kwa miezi ……..
    2. RF, kutumia maziwa ya kopo.
    3. RF, kutumia maziwa ya ng'ombe.
    4. Nyinginezo……………………………………………………

LABOUR AND DELIVERY FORM **FOMU YA MAMA YA KUJIFUNGUA**

NAMBA YA MAMA **M………………………………**

1. Admission in the labour ward. Kulazwa tarehe …………………………………
   Saa aliyolazwa ………………
   Amezalia nyumbani.

2. Diagnosis. Amelazwa kwa ajili ya
   1. Labour uchungu.
   2. Draining Chupa imepasuka.
   3. Antepartum haemorrhage kutoka damu.

3. Did the mother take any ARV during pregnancy? Je mama alitumia ARVs yoyote wakati wa ujauzito?
   0. Hapana 1. Ndiyo.
   Kama ndiyo Aina ………………………..
   Umezitumia kwa muda gani……………….

4. Did the mother take ARV during delivery? Je mama alitumia ARV wakati wa kujifungua?
   0. Hapana 1. Ndiyo.
   Kama ndiyo, aina …………………………

5. Labour onset uchungu ulianza Tarehe………………………………………
   Saa………………….asubuhi/jioni.

6. Membrane rupture, Chupa ilipasuka. Tarehe ……………………………………..
   Saa …………………….asubuhi/jioni.

7. Onset of 1st stage, Hatua ya kwanza ilianza, Muda ………………………………
   Onset 2nd stage, Hatua ya pili ilianza, Muda ………………………………
   Onset 3rd stage, Hatua ya tatu ilianza, Muda ………………………………
   Total duration of labour, Muda tangukuanza uchungu hadi kondo kutoka ……………..

8. *Type delivery Njia aliyojifungua*
   1. *Spontaneous vagina delivery* (SDV).
   2. Breech delivery
   3. Caesarian section (Election…../Emergency…..)

9. Perineum Msamba
   1. Intact Haujachanika.
   2. Tear umechanika.
   3. Episiotomy Aliongezewa njia.

10. mama amezalia wapi?
    1. Hospitali (taja……………………….)
    2. Nyumbani.

Questionnaire - Female

Respondent identification number.............................. Clinic..........................

Gravida (Hii ni mimba ya ngapi?) .... Namba ya watoto waliohai.......

Matatizo katika ujauzito huu? 0.Hapana 1.Ndiyo

L.M.P (Tarehe ya mwisho ya kuona siku)...................... Umri wa mimba..................

Mahali anapotaka kuzalia... Miaka aliyoishi Moshi...........

Jina la anayedodosa .... Tarehe ....

....

1.How old are you? *Je una umri gani?* _____ years/*miaka*

2.What is your religion? *Dini*
- 0.None *Sina*
- 1.Christian *Mkristu*
- 2.Muslim *Mwislamu*
- 3.Traditional believer *Dini ya asili*
- 4.Others *Nyinginezo* ____________________

3.What is your tribe? *Kabila lako?*
- 1.Chagga *Mchaga*
- 2.Pare *Mpare*
- 3.Others *Nyinginezo* ____________________

4.Are you formally employed in which you receive a regular salary? *Je una kazi ambayo unapokea mshahara kila mwisho wa mwezi?*
- 0.No *Hapana* →6
- 1.Yes *Ndiyo* →5

5.What is your job? *Je unafanya kazi gani?*→ 7
- 1.Uniformed job (*Mwanajeshi, Polisi*)
- 2.White collar job (*Nesi, Mwalimu, Daktari*)
- 3.Blue collar job (*Messanger, Msafishaji,, Security guard, Mgambo*)
- 4.Technical job (*Fundi*)
- 5.Other *Nyingine* ____________________

6.How do you generate your cash? *Je unafanya shughuli gani inayokupatia kipato?*
- 1.Farming *Mkulima*
- 2.Small business -vending *Biashara ndogo ndogo*
- 3.Dress making *Kushona*
- 4.Other *Nyinginezo* ____________________

7.What additional activities do you do to subsidise your income? *Je unayo shughuli zaidi ya kazi yako ya sasa inayokuongezea kipato?*
- 0.None *Sina*
- 1.Yes-specify *Ndiyo-taja* ____________________

8.What is your approximate income per month *Kwa kukadiria kipato chako cha mwezi ni kiasi gani?*
- *0.Sina*
- 1.< 30,000 *Tanzanian shs*
- 2.30,000-59,000 *Tanzanian shs*
- 3.60,000-100,000 *Tanzanian shs*
- 4.>100,000 *Tanzanian shs*

9.Level of education (number of years of full time education completed) *Kiwango cha elimu uliyofikia-andika namba ya miaka aliyomaliza*
- 0.No formal education *Sikuwahi kusoma*
- 1.1-7 (Primary level) *Darasa la 1 - 7*
- 2.9 -12 (O level) *Kidato 1–4*
- 3.13-14 ('A' level) *Kidato cha 5-6*
- 4.Higher institutions (Degree, advanced diploma) *Elimu ya juu*

10.House ownership *Je nyumba unayoishi?*
- 1.Own house *Ni yakwenu wenyewe*
- 2.Rent house *Mmepangisha nyumba yote*
- 3. Renting a room/rooms *Mmepangisha chumba/vyumba*
- 4.Living with relatives/my family/parents *Unaishi na ndugu/wazazi/familia yangu*
- 5.Others *Nyinginezo* ____________________

11.Walls *Kuta za nyumba*?
1.Brick *Matofali* 2.Wood *Mbao* 3.Mud *Udongo* 4.*Nyinginezo*_______

11.Toilet facility *Choo unachotumia*
0.No toilet *Hapana* 1.In the house *Kiko ndani ya nyumba*
2.Outside *Kiko nje*

12.Do you share the toilet with other families? *Je mnashirikiana choo na familia nyingine au mnatumia wenyewe?*
0.No *Hapana tunatumia wenyewe* 1.Yes *Ndio tunashirkiana*

13.Do you have electricity? *Nyumbani mnatumia umeme?* 0.No *Hapana* 1.Yes *Ndiyo*

14.Where do you get your water from *Maji yenu ya matumizi mnapata*
1.Tap inside the house *Toka kwenye bomba ndani ya nyumba*
2.Tap outside *Toka kwenye bomba nje ya nyumba*
3.Common supply *Bomba la jumuiya/kijiji*
4.Well *Toka kisimani/ chemchem*

15.Cooking facility *Mara nyingi mnatumia nini kwa kupikia?*
1.Electric stove *Jiko la umeme* 2.Kerosine (Paraffin) *Jiko la mafuta ya taa*
3.Fire Wood *Kuni* 4.Charcoal *Mkaa*

16.Does your family own (multiple responses) *Je mna vitu vifuatavyo nyumbani?*
1.Radio *Redio* 2.Television *Televisheni*
3.Fridge *Friji* 4.Bicycle *Baisikeli*
5.Car *Gari* 6.Telephone *Simu*

17.Do you often travel away from your home? *Je unasafiri mara kwa mara na kwenda nje ya mji/mkoa (unakaa zaidi ya siku moja)?*
0.No *Hapana* 1.Occasionally *Mara chache*
2.Several times a month *Mara nyingi kila mwezi* (mara_____kwa mwezi)

18.What is your marital status *Hali yako ya unyumba kwa sasa ikoje?*
1.Married *Nimeolewa* → 19
2.Cohabiting *Tunaishi pamoja bila ndoa* → 19
3.Single *Sijaolewa* → 20
4.Divorced *Mtalaka (kwa miaka_______________)* → 20
5.Separated *Tumetengana (kwa miaka____________)* → 20
6.Widowed *Mjane (kwa miaka_________________)* → 20

19.How long have you lived together *Je mmeishi pamoja kwa muda gani?* __________ years →21

20.If single, divorced, widowed or separated do you have a steady partner? *Kana hujaolewa, mmetengana, mtalaka, au mjane je una mwenzi wa kila siku?*
0.No *Hapana* → 24, 26 1.Yes *Ndiyo* → 19,21

21.If married/cohabiting or have a steady partner are you in a polygamous marriage or relationship? *Je umeolewa au unaishi na mume mwenye wake wengi?*
0.No *Hapana* 1.Yes *Ndiyo (Namba ya wake ________)*

22.Does your partner have other women outside marriage? *Ukiacha wake, je mume/mwenzi wako ana wanawake wengine nje ya ndoa?*
0.No *Hapana* 1.Yes *Ndiyo* 2.Don't know *Sijui*

23.If married/cohabiting, how do you and your spouse/partner live? *Kama umeolewa/ kuishi pamoja je wewe na mume/mwenzi wako mnaishije?*
1.Together all the time *Pamoja wakati wote*
2.Visits (___times/week) *Ananitembelea (mara_______kwa wiki)*
3.Separate $\geq$ 6 months per year *Tunaishi mbali mbali kwa zaidi ya miezi sita*

24.Have you had other partners before your current spouse/partner? *Je umeshakuwa na mwenzi/ wapenzi wengine kabla ya huyu baba aliyekupa ujauzito wa sasa?*

0.No *Hapana* 1.Yes *Ndiyo* (how many ___________)

25.How old were you when you got married/cohabited for the 1st time? *Ulikuwa na umri gani ulipooewa kwa mara ya kwanza?* ___________ years *miaka*

26.Have you ever experienced any of the following forms of violence from your partner? *Je umeshawahi kufanyiwa yafuatayo na mume/mwenzi wako?*

	No	Yes	No of times
1.Verbal use *Kutukanwa, kusemwa kwa kejeli*	0	1	______
2.Physical assault *Kupigwa au kutandikwa*	0	1	______
3.Sexual abuse *Kulazimishwa kufanya mapenzi bila idhini yako*	0	1	______
4.Other specify *Nyinginezo*______________________			

27.Have you ever been forced to have sex without your consent? *Je umeshawahi kulazimishawa kufanya mapenzi kwa nguvu bila idhini yako au kubakwa?*

0.No *Hapana* 1 Yes. *Ndiyo (na nani*___________)

28.Do you drink alcohol? *Je unakunywa pombe?*

0.No *Hapana* 1.Every day *Kila siku*
2.Once a week *Mara moja kwa wiki* 3.Occasionally *Mara moja moja/ mara chache*

NITAKUULIZA MASWALI MACHACHE KUHUSU MWENZI WAKO/ALIYEKUPA UJAUZITO

29.How old is your present partner *Mume/mwenzi wako ana umri gani*_____ years

30.What is the highest level of formal education he has completed *Kiwango cha elimu aliyofikia?*

0.No formal education *Hakuwahi kusoma* 1.1-7 (Primary level) *Darasa la 1 - 7*
2.9 -12 (O level) *Kidato 1–4* 3.13-14 ('A' level) *Kidato cha 5-6*
4.Higher institutions (Degree, advanced diploma) *Elimu ya juu*

31.What is your partner's main occupation? *Je mume/mwenzi wako anafanya kazi gani?*

1.Uniformed job *(Mwanajeshi, Polisi)*
2.White collar job *(Nesi, Mwalimu, Daktari, Mhasibu)*
3.Blue collar job *(Messanger, Msafishaji,, Security guard, Mgambo)*
4.Technical job *(Fundi wa umeme, magari, kujenga, kushona etc)*
5.Driver.. *Dereva wa malori yaendayo mikoa mingine / dereva wa magari ya hapa mjini*
6.Businessman *Mfanyabiashara*
7.Mkulima *Mkulima*
8.Other *Nyingine*______________________

32.Does your partner travel out of town frequently? *Je mwenzi wako anasafiri mara kwa mara?*

0.No *Hapana* 1.Yes *Ndiyo (Mara*_____ *kwa mwezi)*

33.Is he circumcised? *Je ametahiriwa?*

0.No *Hapana* 1.Yes *Ndiyo*

34.Does your partner drink alcohol *Je mume/mwenzi wako anakunywa pombe?*

0.No *Hapana* 1.Every day *Kila siku*
2.Once a week *Mara moja kwa wiki* 3.Occasionally *Mara moja moja*

b).Does your partner smoke or take any of the following *Je anavuta au kutumia vitu vifuatavyo?*

0.None *Hapana* 1.Cigarattes *Sigara*
2.Marihuana *Bhangi* 3.Khat *Mirungi*

KNOWLEDGE OF STIs/HIV ***NITAKUULIZA MASWALI MACHACHE KUPATA MAWAZO YAKO JINSI UNAVYOELEWA KUHUSU MAGONJWA YA ZINAA NA UKIMWI***

35.Can a pregnant woman pass HIV-1 her baby? (Do not probe) *Je mama mjamzito aliyeathirika na viini vya UKIMWI anaweza kumuambukiza mwanae?*

0.No *Hapana* 1.Yes *Ndiyo* 2.Don't know *Sifahamu*

b).If yes, when does this happen? (Do not probe record all the responses) *Kama ndiyo, inaweza kutokea wakati gani?*

1.Pregnancy *Wakati wa ujauzito* 2.Delivery *Wakati wa kujifungua*
3.Breast-feeding period *Wakati anapomyonyesha mtoto*

36.How do you rate your chances of being infected by HIV *Je ukifikiria, unajiona upo kwenye hatari ya kuambukizwa ugonjwa wa UKIMWI?*

1.No risk *Hapana, sina hatari* → Q 37
2.Small risk *Ndiyo, hatari kidogo* →Q 38
3.Moderate risk *Ndiyo, hatari kiasi* →Q 38
4.High risk *Ndiyo niko kwenye hatari sana* →Q 38
5.Don't know *Sijui*→ *Q 39 Kwa nini?* __________

37.Why do you feel you have no risk? *Kwa nini unaona huna hatari?*

1.I am married *Nimeolewa*
2.Had only one sexual partner in my lifetime *Nimekuwa na mwenzi/mpenzi mmoja tu kwa maisha yangu yote*
3.Currently, I have only one sexual partner *Kwa sasa nina mpenzi/mwenzi mmoja tu*
4.I trust my partner/ he is faithful *Mume wangu ni mwaminifu/ ninamuamini*
5.I always use condom during sex *Ninatumia kondomu kila ninapofanya mapenzi*
6.Others *Nyinginezo*__________

38.Why do you feel you are at risk? *Kwa nini unajiona uko kwenye hatari ya kuambukizwa?*

1.Partner cannot be trusted *Simuamini mume/ mwenzi wangu*
2.Partner has other sexual partners *Mume/mwenzi wangu ana wapenzi wengine/ wengi*
3.I have more than one partner *Nina wapenzi wengi*
4.We don't use condoms *Situmii kondomu wakati ninapofanya mapenzi*
5.I am in polygamous marriage/relationship *Niko kwenye hali ya uke wenza*
6.Others *Nyinginezo*__________

39.Should persons who know they are infected with HIV be entitled to keep this fact a secret from the community where they live or should this information be revealed? *Je unadhanikuwa watu wanaojijua wameathirika na Ukimwi wana haki ya kutomwambia/kutowaambia watu wengine juu ya hali hiyo na iwe ni siri yao au unadhani hali hiyo iwe wazi kwa watu wote wa jumuiya/jamii wanapoishi?*

1.Entitled to keep a secret *Iwe ni siri yao*
2.Should be revealed *Hali hiyo iwe wazi kwa wanajumuiya wote*
3.Do not know *Sijui*

b).Why to either response 1 or 2? *Kwa nini kwa jibu namaba 1 au 2?*
____

40.Should a person infected with HIV tell their partners? *Je unadhani mtu aliyeathirika na UKIMWI amwambie hali hiyo mwenzi/mume wake?*

0.No *Hapana* 1.Yes *Ndiyo* 3.Don't know *Sifahamu*

b).Why to the answer above? *Kwa nini?* __________
____

MASWALI KUHUSU MAMBO YANAYOHUSIANA NA AFYA YAKO

41.Are you circumcised? *Je umetahiriwa au umekeketwa?*

0.No *Hapana* 1.Yes *Ndiyo* (*Umri alipotahiriwa?*______)

b).Who did the circumcision? *Nani aliyekutahiri?* __________

42.If you have a daughter, do you plan to have her circumcised? *Kama una mtoto wa kike, je una mpango wa kumpeleka kutahiriwa/kukeketwa?*

0.No *Hapana* 1.Yes *Ndiyo*

b).Why or why not? *Kwa nini?*__________

Have you ever been treated for the following conditions? ***Je umeshawahi kutibiwa kwa ajili ya hali zifuatazo?***

	No	Yes
43.Abnormal vaginal discharge *Kutoka ute usio wa kawaida*	0	1
44.Genital itching *Kuwashwa sehemu za siri*	0	1
45.Genital ulcers *Vidonda sehemu za siri*	0	1
46.Genital warts *Kuota vinyama katika sehemu za siri*	0	1
47.Painful micturation *Kupata maumivu wakati wa kukojoa*	0	1
48.Pain during sex *Maumivu wakati wa kufanya mapenzi*	0	1
49.Tuberculosis *Kifua kikuu*	0	1
50.Herpes zoster *Herpes zoster-mwilini*	0	1

51.History of small recurring ulcers (herpes)? *Je umeshawahi kupatwa na vidonda vidogovidogo ukeni ambavyo hupona na kurudia mara kwa mara?* 0.No *Hapana* 1.Yes *Ndiyo*

Do you presently have the following symptoms ***Je kwa sasa hivi una dalili zifuatazo?***

	No	Yes
52.Abnormal vaginal discharge? *Kutoka ute usio wa kawaida ukeni*	0	1
53.Genital itching/irritation *Kuwashwa sehemu za siri*	0	1
54.Genital ulcers *Vidonda sehemu za siri*	0	1
55.Genital warts *Kuota vinyama katika sehemu za siri*	0	1
56.Pain during intercourse *Kupata maumivu wakati wa kufanya mapenzi*	0	1
57. Painful micturation *Kupata maumivu wakati unapoenda haja ndogo*	0	1

58.If yes, to any of the above (Q 52-57) *Kama ni ndiyo kati ya swali namba 52-57*

a.*Je uligundua una dalili hizo tangu lini?* ______________________

b. *Je umeshatafuta msaada kwa ajili ya dalili hizo?*

0.*Hapana* → e 1.*Ndiyo* (*Wapi* ______________________)

2.*Ndiyo* (*Matibabu aliyopewa* ______________________)

c.*Kama ulichukua hatua, je ulichukua muda gani toka uone dalili hadi kutafuta msaada?* ______________________

d.*Unaweza kunieleza kwa nini ulichukua zaidi ya siku saba kabla ya kutafuta ushauri?* ______________________

____

e.*Kwa nini hujatafuta huduma au ushauri hadi sasa?* ______________________

f.*Je umeshamueleza mwenzi wako ju ya dalili hizi?* 0.*Hapana* 1.*Ndiyo*

g. *Je uliendelea kukutana kimwili na mwenzi wako wakati una dalili ulizotaja hapo juu?* 0. *Hapana* 1.*Ndiyo*

h.*Je mlitumia kondomu wakati wa kufanya mapenzi?* 0.*Hapana* 1. *Ndiyo*

NITAKUULIZA MASWALI MACHACHE KUHUSU KUKUTANA KIMWILI

59.How old were you when you had sexual intercourse for the first time? *Ulikuwa na umri gani ulipokutana kimwili mara ya kwanza? Miaka* ______ years

60.Was it voluntary/ did you want to do it? *Je ulitaka kufanya kitendo hicho au ulilazimishwa?*

0.No *Hapana, sikutaka nililazimishwa* 1.Yes *Ndiyo, nilitaka*

61.Did you use a condom during your first sexual encounter? *Je ulitumia kondomu ulipokutana kimwili kwa mara ya kwanza?*

0.No *Hapana* 1.Yes *Ndiyo*

62.During the last 12 months have you had any other sexual partner/s besides your regular partner? *Je ni watu wangapi tofauti ambao umekutana nao kimwili kwa kipindi cha miezi 12 iliyopita ukiacha mwenzi/mume wako?*

0.No *Hakuna sijakutana na mwingine* 1.Yes *Ndiyo, idadi*__________

63.How many sexual partners have you had in your life time including your current partner? *Je kwa ujumla ukimweka na mume/mwenzi wako wa kila siku, umeshakuwa na wenzi /wapenzi wangapi tangu ulipokutana kimwili kwa mara ya kwanza hadi sasa?*

1.One *Mmoja tu* 2.Two *Wawili*
3.Three or more *Watatu na zaidi, idadi*______

64.Have you ever used a condom? *Je umewahi kutumia kondomu/mpira wa kuuvisha uume wakati wa kukutana kimwili?*

0.No *Hapana* 1.Yes *Ndiyo*

NITAKUULIZA MASWALI MCHACHE KUHUSU MAMBO YA UZAZI

65.How old were you when you got pregnant the the 1st time? *Je ulikuwa na umri gani ulipopata ujauzito kwa mara ya kwanza? Miaka*______years

66.Number of living children *Una jumla ya watoto wangapi walio hai?* ____ *(Kama mimba ya kwanza* → *75)*

**Have you ever had pregnancy that; *Umeshawahi kupata ujauzito ambao***	**No**	**Yes,**	**No of times**
67.Ended in confirmed spontaneous abortion *Mimba ilitoka yenyewe*	0	1	____
68.Ended in stillbirth *Mtoto alizaliwa amekufa*	0	1	____

**Have you ever given birth to a child who was** **Umeshawahi kuzaa mtoto ambaye**			
69.A premature baby *Amezaliwa kabla ya siku/njiti*	0	1	____
70.Below 2500 grams at birth *Aliyekuwa na uzito wa gramu 2500 au chini?*	0	1	____
71.Born alive but later died before the age of one year? *Alikuwa mtoto hai, lakini akafariki kabla ya kufikia umri wa mwaka mmoja?*	0	1	____

72.Where did you deliver your last baby? *Je ulimzalia wapi mtoto wako wa mwisho?*

0.Not applicable 1.Hospital *hospitali* 2.Home *nyumbani*

b).If at home, why? *Kwa nini ulijifungulia nyumbani?*__________

73.Age of the last born *Umri wa mtoto wa mwisho Miaka*________

74.Do all your children have the same father *Je watoto wako wote wana baba mmoja?*

0.No *Hapana* 1.Yes *Ndiyo*

75.Have you ever practised any family planning method in your lifetime? *Je umeshawahi kutumia njia yoyote ya uzazi wa majira/mpango?*

0.No *Hapana* 1.Pills *Vidonge*
2.Injection *Sindano* 3.IUD *Kitanzi*
4.Condoms *Kondomu* 5.Others__________

76.Was the current pregnancy planned? *Mimba hii uliitaka au uliipata kwa bahati mbaya?*

0.No *Hapana ilikuwa bahati mbaya* 1.Yes *Ndiyo niliitaka*

Concerning the current pregnancy *Katika ujauzito huu*

77.Have you received haematenics *umeshawahi kupewa vidonge vya chuma vya kuongeza damu?*___
78.Have you received prophylactic antimalarials *umeshawahi kupewa dawa za kukinga malaria?*___
79.Have you been treated for malaria? *Umeshatibiwa kwa ajili ya malaria?* ______

80.Are you abstaining from sex now that you are pregnant? *Je umeacha kukutana kimwili na mwenzi wako kwa sasa kwa ajili ya ujauzito?* 0.No *Hapana* 1.Yes *Ndiyo*

b).If yes, why? *Kwa nini?*__________

COUPLE COMMUNICATION ***MAJADILIANO KATI YA WATU WANAOISHI PAMOJA***

81.Have you ever discussed with your partner about matters relating *to Je umeshawahi kujadiliana na mume/mwenzi wako kuhusu mambo yafuatayo?*

	0.No *Hapana*	1.Yes *Ndiyo*
1.Condom use *Kuhusu kutumia kondomu*	0	1
2.Contraceptives/family planning *Uzazi wa mpango*	0	1
3.Desired number of children *Namba ya watoto unaowataka*	0	1
4.Sexual satisfaction/sexuality *Kukuridhisha kimapenzi*	0	1
5.About HIV *Kuhusu ukimwi*	0	1
6.About STDs *Kuhusu magonjwa ya zinaa*	0	1

82.If we found you are HIV-1 positive, who will you want to inform/discuss the results with? *Je tukikupima na kukuta na UKIMWI, ungependa kushirikiana majibu yako na nani?*__________

(Kama mwenzi hajatajwa nenda swali la 83 a, b, c

83.If the partner is not among them, will she be able to inform her partner? *Kama mume/mwenzi hakutajwa, je tukikupima na kukuta na ukimwi utaweza kumwambia/ kumjulisha?*

0.No *Hapana* 1.Yes *Ndiyo*

b).If no, why would you not inform him? (multiple responses) *Kama hapana, kwanini?*

1.Fear of being blamed for bringing the infection home *Naogopa atanilaumu ni mimi nimeleta ugonjwa katika ndoa*
2.Fear of violence (verbal/phyisical abuse) *Naogopa anaweza akanipiga au kunitukana*
3.Fear of being divorced *Naogopa ataniacha*
4.Fear that he will tell his relatives *Naogopa atawaambia ndugu zake*
5.Other specify *Nyinginezo*__________

c)If no, how will you approach the question of sex with him? *Kama hutamjulisha itakuwaje kuhusu suala la kukutana kimwili?*__________

Swali Lipya → 84.Umeshawahi kupima ukimwi au hii ni mara ya kwanza? 1.Mara ya kwanza 2.Nimeshapima

END OF QUESTIONNAIRE ***MWISHO WA MASWALI***

THANK YOU FOR ANSWERING THESE QUESTIONS ***ASANTE KWA KUJIBU MASWALI***

Muulizaji kumbuka kumkumbusha mama juu ya yafuatayo

- Rudia kuhusu STDs/HIV na jinsi ya kujikinga na mpe elimu katika yale maswali unyoona mama hakuwa na elimu juu yake.
- Mkumbushe atapimwa na daktari na vipimo vya mkojo, damu na ukeni vitachukuliwa
- Ya kuwa majibu yote ni siri hivyo itabidi aje kurudia majibu mwenyewe

PHYSICAL EXAMINATION

1.Weight (*kg*) ☐☐ 2.Height (*cm*) ☐☐☐ 3.MUAC ☐☐☐

4.Blood pressure ☐☐☐ / ☐☐

5.General appearance ☐ 1.*Looks well* ☐ 2. *Looks sick* ☐ 3.*Pale conjuctiva/mucosa*

6. Nutrition status
☐ 1. *Good* ☐ 2. *Poor*

7.Lymphadenopathy (neck, axilla) ☐ *0. No* ☐ *1. Yes*

8.Oedema ☐ *0.No* ☐ *1.Yes*

9.Skin ☐ *1. Normal* ☐ *2. Rashes* ☐ *3. Ulcers*

10.Eyes/ ENT ☐ *1. Normal* ☐ *2. Eye discharge* ☐ *3. Ear discharge*

11.Throat ☐ *1. Normal* ☐ *2. Inflamed* ☐ *3.Tonsilar enlargement*

12.Breasts ☐ *1.Normal* ☐ *2. Abscess* ☐ *3.Ulcers/cracks* ☐ *4. Lumps*

13.Oral thrush ☐ *0. No* ☐ *1.Yes*

14.Respiratory
☐ *1. Normal* ☐ *2. Cyanosed* ☐ *3. Respiratory wheeze*
☐ *Air entry good* ☐ *Air entry poor*

15.Genitalia
☐ *0.Not circumcised* ☐ *1.Partial clitoridectomy* ☐ *2.Total clitoridectomy*
☐ *3.Clitoridectomy+excised labia minora* ☐ *4.Infibulation*

Vulva ☐ *1 normal* ☐ *2.Ulcers(single/multiple)* ☐ *3.Warts*

Vagina ☐ *0.normal* ☐ *1.Yellow discharge* ☐ *2.Thick white discharge*
☐ *3.Greyish discharge* ☐ *4.Warts*

Cervix ☐ *1. Normal cervix* ☐ *2. Cervical discharge (swab test)* ☐ *3.Cervical ectopy*
☐ *4.Ulcer* ☐ *5.Easily induced bleeding* ☐ *6.Cervical cancer*

PH ☐ *1.≤4.5* ☐ *2.> 4.5*

Whiff test ☐ *1.Negative* ☐ *2.Positive*

Questionnaire - Female

Respondent identification number:………………………   Clinic……………………………

Gravida (Hii ni mimba ya ngapi?) ……………………....   Namba ya watoto waliohai.......

Matatizo katika ujauzito huu?   0.Hapana   1.Ndiyo

L.M.P (Tarehe ya mwisho ya kuona siku)………………..   Umri wa mimba..................

Mahali anapotaka kuzalia………………………………   Miaka aliyoishi Moshi………

Jina la anayedodosa ……………………………...........   Tarehe ………………………

………………………………………………………………………………………………

1.Je una umri gani? __________miaka   Kabila - - - -

2. Dini   0.Sina   1. Mkristu   2.Mwislamu   3. Dini ya asili

3. Je umeajiriwa na unapokea mshahara mwisho wa mwezi   0.Hapana   1.Ndiyo (Kazi____________

4.Kama hujaajiriwa, unafanya shughuli gani inayokupatia kipato? (Taja________________________

5.Kwa kukadiria kipato chako cha mwezi ni kiasi gani?
- 0.Sina
- 1.< 30,000 Tanzanian shs
- 2.30,000-59,000 Tanzanian shs
- 3.60,000-100,000 Tanzanian shs
- 4.>100,000 Tanzanian shs

6.Kiwango cha elimu uliyofikia-andika namba ya miaka aliyomaliza
- 0.Sikuwahi kusoma
- 1. (Primary level) Darasa la 1 - 7
- 2. (O level) Kidato 1–4
- 3. ('A' level) Kidato cha 5-6
- 4.Higher institutions (Degree, advanced diploma) Elimu ya juu

7.Hali yako ya unyumba kwa sasa ikoje?
- 1.Nimeolewa (Ni bwana wa wangapi?__________________) → 8
- 2. Tunaishi pamoja bila ndoa (Ni bwana wa wangapi?__________________) → 8
- 3. Sijaolewa → 9
- 4.Tumetengana (kwa miaka___________) /Mjane (kwa miaka_________________)→ 9

8.Je mmeishi pamoja kwa muda gani?   Kwa miaka ____________________ →10

9.Kana hujaolewa, mmetengana, au mjane je una mwenzi wa kila siku?
- 0.Hapana → 13
- 1.Ndiyo → 8,10

10.Je umeolewa au unaishi na mume mwenye wake wengi?   0.Hapana   1.Ndiyo (*Namba ya wake*__

11.Je mume/mwenzi wako ana wanawake wengine nje ya ndoa?
- 0.Hapana
- 1.Ndiyo
- 2. Sijui

12.Kama umeolewa/ kuishi pamoja je wewe na mume/mwenzi wako mnaishije?
- 1.Pamoja wakati wote
- 2.Ananitembelea (mara_______kwa wiki)
- 3. Tunaishi mbali mbali kwa zaidi ya miezi sita
- 4.Nyingine (Taja_________________)

13.Je umeshakuwa na mwenzi/wapenzi wengine kabla ya huyu baba aliyekupa ujauzito wa sasa?
- 0.Hapana
- 1.Ndiyo (*Wangapi* ___________)

14.Ulikuwa na umri gani ulipoolewa kwa mara ya kwanza?   Miaka______________

15.Je umeshawahi kulazimishawa kufanya mapenzi kwa nguvu bila idhini yako au kubakwa?
- 0.Hapana
- 1 Ndiyo (na nani______________________)

16.Je unakunywa pombe?
- 0.No Hapana
- 1.Ndiyo (Kila siku /Mara moja kwa wiki/Mara moja moja)

17.Mume/mwenzi wako ana umri gani? Miaka____________

18.Kiwango cha elimu aliyofikia?
0.Hakuwahi kusoma
1. (Primary level) Daraşa la 1 - 7
2. (O level) Kidato 1–4
3. ('A' level) Kidato cha 5-6
4.Higher institutions (Degree, advanced diploma) Elimu ya juu

19.Je mume/mwenzi wako anafanya kazi gani? ______________________________

KNOWLEDGE OF STIs/HIV

20. Je mama mjamzito aliyeathirika na viini vya UKIMWI anaweza kumuambukiza mwanae?
0.Hapana 1. Ndiyo 2.Sifahamu

b).Kama ndiyo, inaweza kutokea wakati gani? *(Zungushia majibu yote anayotaja)*
1.Wakati wa ujauzito 2.Wakati wa kujifungua 3.Wakati anapomyonyesha mtoto

21.How do you rate your chances of being infected by HIV Je ukifikiria, unajiona upo kwenye hatari ya kuambukizwa ugonjwa wa UKIMWI?

1.No risk	Hapana, sina hatari → Q 22	
2.Small risk	Ndiyo, hatari kidogo →Q ~~22~~ 23	
3.Moderate risk	Ndiyo, hatari kiasi →Q 23	
4.High risk	Ndiyo niko kwenye hatari sana →Q 23	
5.Don't know	Sijui→ Q 24 Kwa nini?______________________	

22.Kwa nini unaona huna hatari? ______________________________

23.Kwa nini unajiona uko kwenye hatari ya kuambukizwa? ______________________________

24.Je umetahiriwa au umekeketwa? 0.Hapana 1.Ndiyo (*Umri alipotahiriwa?*__________)

b).Nani aliyekutahiri? ______________________________

25.Ukijaliwa kupata mtoto wa kike, je unatampeleka kutahiriwa? 0. Hapana 1. Ndiyo

b).Kwa nini kwa jibu atakalotoa? ______________________________

**Je umeshawahi kutibiwa kwa ajili ya hali zifuatazo?**		**No**	**Yes**
26.Abnormal vaginal discharge	Kutoka ute usio wa kawaida	0	1
27.Genital itching	Kuwashwa sehemu za siri	0	1
28.Genital ulcers	Vidonda sehemu za siri	0	1
29.Genital warts	Kuota vinyama katika sehemu za siri	0	1
30.Painful micturation	Kupata maumivu wakati wa kukojoa	0	1
31.Pain during sex	Maumivu wakati wa kufanya mapenzi	0	1
32.Syphilis	Kaswende	0	1
33.Herpes zoster	Herpes zoster-mwilini	0	1

34. **Je umeshawahi kupatwa na vidonda vidogovidogo ukeni ambavyo hupona na kurudia mara kwa mara?** **0.Hapana** **1. Ndiyo**

**Je kwa sasa hivi una dalili zifuatazo?**		**No**	**Yes**
35.Abnormal vaginal discharge?	Kutoka ute usio wa kawaida ukeni	0	1
36.Genital itching/irritation	Kuwashwa sehemu za siri	0	1
37.Genital ulcers	Vidonda sehemu za siri	0	1
38.Genital warts	Kuota vinyama katika sehemu za siri	0	1
39.Pain during intercourse	Kupata maumivu wakati wa kufanya mapenzi	0	1
40. Painful micturation	Kupata maumivu wakati unapoenda haja ndogo	0	1

41.Je unashafisha ndani ya uke? 0.Hapana 1.Ndiyo (*Unatumia nini kuoshea?*____________
*Unakuosha mara ngapi kwa siku?*________________

42. Ulikuwa na umri gani ulipokutana kimwili mara ya kwanza? Miaka__________________

43.Je ulitaka kufanya kitendo hicho au ulilazimishwa?
0.Hapana, sikutaka nililazimishwa 1.Ndiyo, nilitaka

44.Je ulitumia kondomu ulipokutana kimwili kwa mara ya kwanza? 0.Hapana 1.Ndiyo

45.Je ni watu wangapi tofauti ambao umekutana nao kimwili kwa kipindi cha miezi 12 iliyopita ukiacha mwenzi/mume wako?
0.Hakuna sijakutana na mwingine 1.Ndiyo, idadi____________________

46.Je kwa ujumla ukimweka na mume/mwenzi wako wa kila siku, umeshakuwa na wenzi /wapenzi wangapi tangu ulipokutana kimwili kwa mara ya kwanza hadi sasa? Idadi____________________

47.Je umewahi kutumia kondomu/mpira wa kuuvisha uume wakati wa kukutana kimwili?
0.No Hapana 1.Yes Ndiyo

48.Je ulikuwa na umri gani ulipopata ujauzito kwa mara ya kwanza? Miaka____________

49.Una jumla ya watoto wangapi walio hai? _____(Kama mimba ya kwanza→ 57)

**Umeshawahi kupata ujauzito ambao**	**No**	**Yes**	**No of times**
50.Mimba ilitoka yenyewe / kuharibika (abortion)	0	1	_____ (*Ilitoka na miezi*___)
51.Mtoto alizaliwa amekufa (stillbirth)	0	1	_____

Umeshawahi kuzaa mtoto ambaye

52.A premature baby	Amezaliwa kabla ya siku/njiti	0	1	________
53.Below 2500 grams at birth	Aliyekuwa na uzito wa gramu 2500 au chini?	0	1	________
54.Alikuwa mtoto hai, lakini akafariki kabla ya kufikia umri wa mwaka mmoja?		0	1	________

55.Umri wa mtoto wa mwisho Miaka________________________________

56.Je watoto wako wote wana baba mmoja? 0.Hapana 1.Ndiyo

57.Je umeshawahi kutumia njia yoyote ya uzazi wa majira/mpango?
0.No Hapana 1.Ndiyo (*Taja*__________________________

58.Mimba hii uliitaka au uliipata kwa bahati mbaya?
0.Hapana ilikuwa bahati mbaya 1. Ndiyo niliitaka

59.Je huko majumban,i watu wenye ndugu wenye ukimwi wanaishije nao?____________________

____

60. Je tukikupima na kukuta na UKIMWI, ungependa kushirikiana majibu yako na nani?____________

(Kama mwenzi hakutajwa nenda swali la 61)

61.Kama mume/mwenzi hakutajwa, je tukikupima na kukuta na ukimwi utaweza kumwambia/ kumjulisha? 0.Hapana 1.Ndiyo

b).Kama hapana, kwanini? (*Zungushia zote anazotaja*)
1.Naogopa atanilaumu ni mimi nimeleta ugonjwa katika ndoa
2.Naogopa anaweza akanipiga au kunitukana
3.Naogopa ataniacha
4.Naogopa atawaambia ndugu zake
5.Sababu nyinginezo, taja ____

c) Kama hutamjulisha itakuwaje kuhusu suala la kukutana kimwili? __________________________

62. Umeshawahi kupima ukimwi au hii ni mara ya kwanza? 1.Mara ya kwanza 2.Nimeshapima

Muulizaji kumbuka kumkumbusha mama juu ya yafuatayo

- Rudia kuhusu STDs/HIV na jinsi ya kujikinga na mpe elimu katika yale maswali unyoona mama hakuwa na elimu juu yake.
- Mkumbushe atapimwa na daktari na vipimo vya mkojo, damu na ukeni vitachukuliwa
- Ya kuwa majibu yote ni siri hivyo itabidi aje kurudia majibu mwenyewe

PHYSICAL EXAMINATION

1.Weight (kg) ☐☐ 2.Height (cm) ☐☐☐ 3.MUAC ☐☐☐

4.Blood pressure ☐☐☐ / ☐☐

5.General appearance ☐ 1.Looks well ☐ 2. Looks sick ☐ 3.Pale conjuctiva/mucosa

6. Lymphadenopathy (neck, axilla) ☐ 0. No ☐ 1. Yes

7.Oedema ☐ 0.No ☐ 1.Yes

8.Skin ☐ 1. Normal ☐ 2. Rashes ☐ 3. Ulcers

9.Breasts ☐ 1.Normal ☐ 2. Abscess ☐ 3.Ulcers/cracks ☐ 4. Lumps

10.Oral thrush
☐ 0. No ☐ 1.Yes

11.Genitalia
☐ **0.Not circumcised** ☐ **1.Partial clitoridectomy** ☐ **2.Total clitoridectomy**
☐ **3.Clitoridectomy+excised labia minora** ☐ **4.Infibulation**

Vulva ☐ 1 normal ☐ 2.Ulcers(single/multiple) ☐ 3.Warts

Vagina ☐ 0.normal ☐ 1.Yellow discharge ☐ 2.Thick white discharge
☐ 3.Greyish discharge ☐ 4.Warts

Cervix ☐ 1. Normal cervix ☐ 2. Cervical discharge (swab test) ☐ 3.Cervical ectopy
☐ 4.Ulcer ☐ 5.Easily induced bleeding ☐ 6.Cervical cancer

PH ☐ 1.≤4.5 ☐ 2.> 4.5

Whiff test ☐ 1.Negative ☐ 2.Positive

QUESTIONNAIRE FUPI inaendelea.........

63. Je kabla ya ujauzito huu, ulikuwa unakula mara ngapi kwa siku ?____________________

64. Naomba unitajie vyakula ulivyokuwa unakula kwa kawaida tangu unapoamka asubuhi hadi usiku______

Asubuhi hadi saa sita mchana: ____________________

____

Mchana hadi saa kumi na mbili jioni: ____________________

____

Jioni/Usiku: ____________________

____

65. Je kwa sasa hivi na ujauzito huu, unakula mara ngapi kwa siku ?____________________

66. Naomba unitajie vyakula unayokula kwenye ujauzito huu, tangu unapoamka asubuhi hadi usiku ______

Asubuhi hadi saa sita mchana: ____________________

____

Mchana hadi saa kumi na mbili jioni: ____________________

____

Jioni/Usiku: ____________________

____

QUESTIONNAIRE FUPI inaendelea.........

63. Je kabla ya ujauzito huu, ulikuwa unakula mara ngapi kwa siku ?____________________

64. Naomba unitajie vyakula ulivyokuwa unakula kwa kawaida tangu unapoamka asubuhi hadi usiku______

Asubuhi hadi saa sita mchana: ____________________

____

Mchana hadi saa kumi na mbili jioni: ____________________

____

Jioni/Usiku: ____________________

____

65. Je kwa sasa hivi na ujauzito huu, unakula mara ngapi kwa siku ?____________________

66. Naomba unitajie vyakula unayokula kwenye ujauzito huu, tangu unapoamka asubuhi hadi usiku ______

Asubuhi hadi saa sita mchana: ____________________

____

Mchana hadi saa kumi na mbili jioni: ____________________
